# The NRPD1 N-terminus contains a Pol IV-specific motif that is critical for genome surveillance in Arabidopsis

**DOI:** 10.1093/nar/gkz618

**Published:** 2019-08-02

**Authors:** Laura Ferrafiat, David Pflieger, Jasleen Singh, Michael Thieme, Marcel Böhrer, Christophe Himber, Aude Gerbaud, Etienne Bucher, Craig S Pikaard, Todd Blevins

**Affiliations:** 1 Institut de Biologie Moléculaire des Plantes, CNRS, Université de Strasbourg, F-67084 Strasbourg, France; 2 Howard Hughes Medical Institute, Indiana University, Bloomington, IN 47405, USA; 3 Department of Biology, Indiana University, Bloomington, IN 47405, USA; 4 Botanisches Institut, Universität Basel, CH-4056 Basel, Switzerland

## Abstract

RNA-guided surveillance systems constrain the activity of transposable elements (TEs) in host genomes. In plants, RNA polymerase IV (Pol IV) transcribes TEs into primary transcripts from which RDR2 synthesizes double-stranded RNA precursors for small interfering RNAs (siRNAs) that guide TE methylation and silencing. How the core subunits of Pol IV, homologs of RNA polymerase II subunits, diverged to support siRNA biogenesis in a TE-rich, repressive chromatin context is not well understood. Here we studied the N-terminus of Pol IV’s largest subunit, NRPD1. Arabidopsis lines harboring missense mutations in this N-terminus produce wild-type (WT) levels of NRPD1, which co-purifies with other Pol IV subunits and RDR2. Our *in vitro* transcription and genomic analyses reveal that the NRPD1 N-terminus is critical for robust Pol IV-dependent transcription, siRNA production and DNA methylation. However, residual RNA-directed DNA methylation observed in one mutant genotype indicates that Pol IV can operate uncoupled from the high siRNA levels typically observed in WT plants. This mutation disrupts a motif uniquely conserved in Pol IV, crippling the enzyme's ability to inhibit retrotransposon mobilization. We propose that the NRPD1 N-terminus motif evolved to regulate Pol IV function in genome surveillance.

## INTRODUCTION

Genome surveillance pathways have evolved in eukaryotes to limit deleterious mutations by recognizing and silencing transposable elements (TEs). Because TEs vary in sequence and replication mechanism, animals and plants use RNA silencing to guide repressive effects to TEs (1–3). A specialized non-coding RNA machinery, involving multisubunit RNA polymerases IV and V (Pol IV and Pol V), is the key nuclear pathway targeting TEs in plants (4,5). To initiate this RNA-directed DNA methylation (RdDM) process, Pol IV transcribes TEs and repeat-associated genes into primary transcripts that are templates for RNA-DEPENDENT RNA POLYMERASE 2 (RDR2) (6). RDR2 synthesizes double-stranded RNAs (dsRNAs) that are processed into 24 nt small interfering RNAs (siRNAs) by DICER-LIKE 3 (DCL3) (6–10). These siRNAs are loaded onto ARGONAUTE 4 (AGO4)-clade proteins (11,12) and resulting AGO4–siRNA complexes appear to find TEs via base-pairing of the siRNA guide to nascent Pol V transcripts in chromatin (13,14). The *de novo* cytosine methyltransferase DRM2 is then recruited, resulting in TE methylation, repressive histone modifications and silencing (15).

In heat-stressed *Arabidopsis thaliana* (Arabidopsis), Pol IV-RdDM inhibits the replication and transgenerational mobilization of *ATCOPIA78/ONSEN* retrotransposons (16–18). To accomplish this, DOMAINS REARRANGED METHYLTRANSFERASE 2 (DRM2) methylates cytosines in CG, CHG and CHH sequence contexts, where H is A, C or T. However, most TEs are also silenced by DNA methylation and chromatin modification systems that are siRNA-independent (19–21). METHYLTRANSFERASE 1 (MET1) maintains CG methylation while influencing CHG and CHH methylation (22,23); CHROMOMETHYLASE 3 (CMT3) maintains CHG methylation while reinforcing CHH methylation at certain targets (24); and CHROMOMETHYLASE 2 (CMT2) methylates a subset of CHG and CHH sites, typically in the bodies of longer TEs (25,26). Biochemical feedback loops link methylcytosine maintenance to repressive histone modifications (notably, histone 3 lysine 9 dimethylation; H3K9me2), reinforcing the silent chromatin states that inhibit TE activity (27,28). Moreover, the nucleosome remodeler DDM1 and histone deacetylase HDA6 facilitate a significant fraction of DNA methylation in plants (27). Because of these overlapping silencing machineries, the role of Pol IV in TE repression is most evident in MET1, DDM1 or HDA6-deficient backgrounds, where RdDM compensates for losses in H3K9me2 and CG methylation to limit TE reactivation (2,19–21,29).

Pol IV is a twelve-subunit enzyme that evolved in plants as a specialized form of eukaryotic RNA polymerase II (Pol II) (30). Pol IV localization to TEs is mediated by the SAWADEE HOMEODOMAIN HOMOLOGUE 1 (SHH1) protein, which recognizes H3K9me2 and unmethylated H3K4; about 50% of Pol IV-dependent siRNA clusters depend on SHH1 (31). In addition, the SNF domain-containing CLASSY proteins (CLSY1/2/3/4) facilitate locus-specific methylation via their interactions with Pol IV and SHH1 (32–35). Little is known about how the core domains of Pol IV have evolved to support its unique function in silencing. The insensitivity of Pol IV to α-amanitin, its elevated error rate, its physical coupling to RDR2, and the Pol IV-RDR2 complex's short dsRNA products all distinguish Pol IV transcription from Pol II transcription (8–10,33,36). Nevertheless, similar to Pol IV loss-of-function, inhibiting Pol II boosts the activity of certain retrotransposons because Pol II generates precursors for siRNAs that trigger ‘non-canonical’ RdDM (18,37–39). Many questions thus remain about how the activities of Pol II, Pol IV and Pol V are differentiated, balanced and regulated *in vivo* to prevent TE proliferation (36,39–41).

Screens for RNA interference factors using transgene-encoded silencers, like the potato virus X amplicon system or dsRNA directed against endogenous genes, have yielded numerous *pol IV* mutations in Arabidopsis (32,42–45). However, past molecular analyses of Pol IV *in vivo* function have typically focused on null alleles of its major subunits (NRPD1 and NRPD2), which either block Pol IV accumulation altogether or abolish Pol IV enzymatic activity by destroying the RNA polymerase active site (21,31,44,46–50). In addition, null alleles of the NRPD4/E4 subunit, which functions in both Pol IV and Pol V, were found to reduce siRNA production from a subset of Pol IV-dependent loci (45).

Here we studied missense mutations in the N-terminus of Arabidopsis NRPD1 that disrupt Pol IV function by changing amino acids remote from the RNA polymerase active site. The mutants do not disrupt Pol IV subunit assembly or RDR2 association but show partial derepression of TEs. Small RNA sequencing and methylome analyses of the N-terminus mutants indicate that Pol IV can mediate RdDM at subfeatures of TEs without generating the high siRNA levels typical of wild-type (WT) plants. Phylogenetic analysis of one mutation's context revealed an N-terminal motif, uniquely conserved in Pol IV, which facilitates 24 nt siRNA production and CHH methylation across the length of TEs. Disrupting this motif cripples the ability of Pol IV to inhibit *ONSEN* retrotransposon mobilization. We propose that this NRPD1 subdomain evolved to facilitate RdDM and genome surveillance, illuminating its potential role in regulating Pol IV transcription more generally.

## MATERIALS AND METHODS

### Plant materials

*Arabidopsis thaliana* (Arabidopsis) T-DNA insertion mutants *nrpd1-3, nrpe1-11* and *nrpd/e2-2* were described in (46,51). The *nrpd1* point mutations were obtained in an EMS screen using the *SUC2::IR-SUL* silencing reporter transgene in a *35S::DCL4* background (SucSul D4). Each *nrpd1* point mutant was backcrossed to WT Col-0 to eliminate *35S::DCL4* and restored to a homozygous state prior to molecular analyses shown in Figures 3, 5 and 6. The *SUC2::IR-SUL* transgene was recovered in a homozygous state after backcrossing because it is linked to the *NRPD1* locus. Mutants for DNA methyltransferases (*drm2-2, cmt3-11*) or RNA-dependent RNA polymerases (*rdr2-2, rdr6-11*) were obtained from the Arabidopsis Biological Resource Center. *RDR2-FLAG rdr2-2* lines were generated by recombining a pENTR-RDR2 genomic clone into pEarleyGate302 and complementing the *rdr2-2* null mutant by Agrotransformation. To enable Pol IV-RDR2 co-immunopurification and Pol IV *in vitro* transcription experiments in *nrpd1* mutant backgrounds, *RDR2-FLAG rdr2-2* was crossed to each of the *nrpd1-47* through *nrpd1-51* mutants. F2 progeny were then selected in which each *nrpd1* allele was homozygous and RDR2-FLAG was robustly expressed. Double mutants of each *nrpd1* mutant with *drm2-2, cmt3-11, nrpe1-11*and*rdr6-11* were obtained by crossing the corresponding lines and selecting homozygous F2 progeny by polymerase chain reaction (PCR)-based genotyping.

### Heat stress and retrotransposon detection

Surface-sterilized seed from Arabidopsis control (WT Col-0, WT SucSul) and *nrpd1* mutant lines were grown axenically in a Sanyo MLR-350 chamber on solid 0.5X MS medium (1% sucrose, 0.5% Phytagel (Sigma), pH 5.8) under long day conditions (16 h light) at 24°C (day) and 22°C (night). After one week of growth, plants were either exposed to a control stress (CS, 24 h at 6°C followed by 24 h at control conditions) or to an acute heat stress (24 h at 6°C followed by 24 h at 37°C). These treatments, tissue sampling and qPCRs to measure *ONSEN*-copy numbers were conducted as described previously (16,18) (see Supplementary Table S1 for primer sequences).

### Antibodies

**Figure 1.**
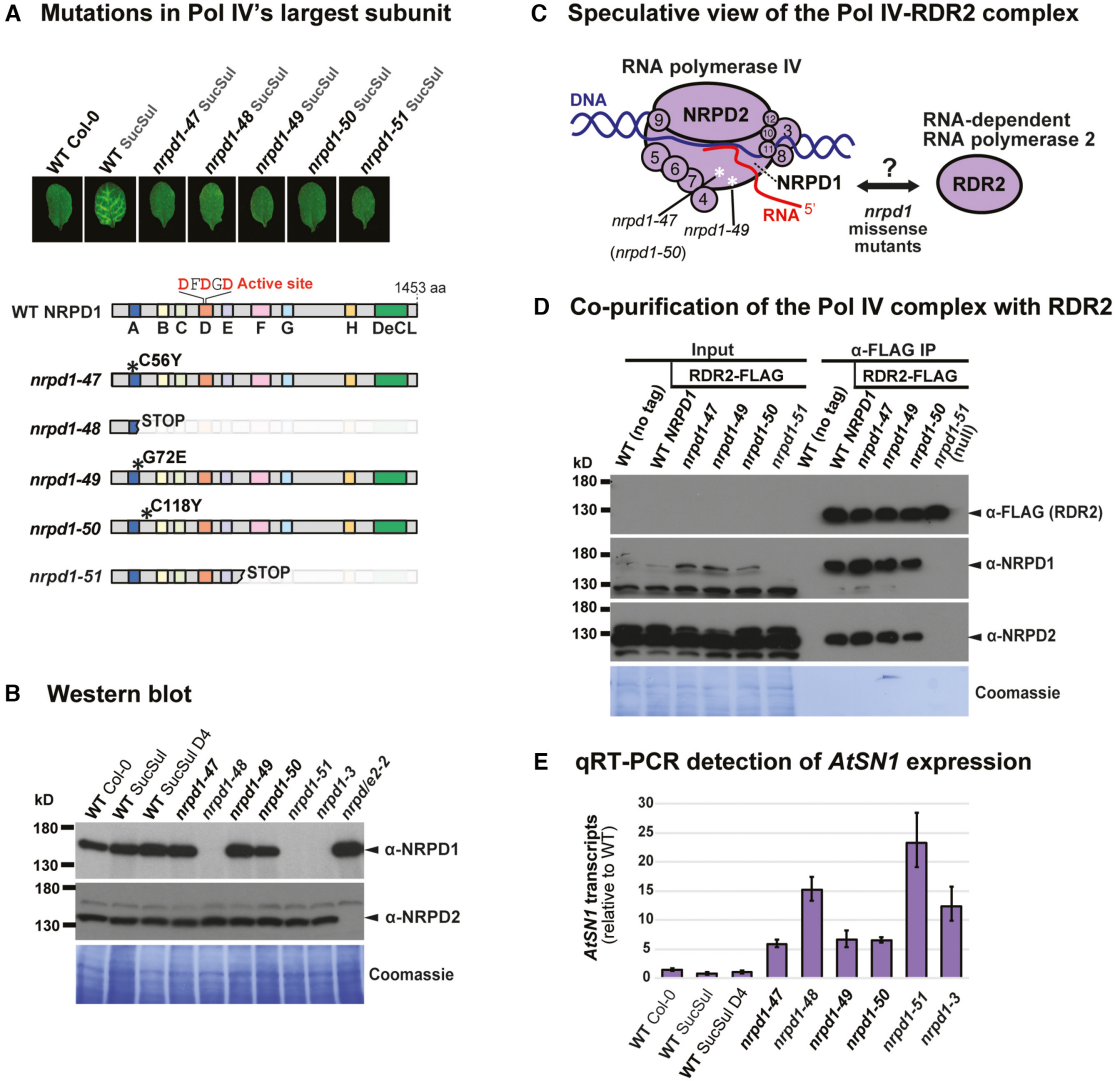
N-terminal missense alleles in NRPD1 disrupt the *in vivo* function of Pol IV-RDR2. (**A**) Above: Rosette leaves are shown from WT Col-0, WT SucSul and from *nrpd1-47, nrpd1-48, nrpd1-49, nrpd1-50* and *nrpd1-51* mutants (all in the SucSul background). Below: Diagram showing the position and predicted effect of the five mutations on NRPD1, Pol IV’s largest subunit. Denoted along the length of NRPD1 are the evolutionarily conserved Domains A to H of multisubunit RNA polymerase largest subunits (46,57). The ‘Defective in Chloroplasts and Leaves’ (DeCL) domain is specific to Pol IV and Pol V and not found outside plants. (**B**) Western blot detection of Pol IV subunit accumulation in crude protein isolated from inflorescences of WT controls (Col-0, SucSul and SucSul D4), *nrpd1* mutants and the *nrpd/e2* mutant. Antibodies used here were raised against peptides from the NRPD1 C-terminus or the NRPD2 N-terminus, respectively. (**C**) Speculative view of the Pol IV complex (modified from (70)) showing estimated positions (*) of the amino acid substitutions *nrpd1-47* (C56Y) and *nrpd1-49* (G72E) inferred from alignment of Arabidopsis NRPD1 to yeast RPB1 and the related Pol II structure (57). (**D**) WT and mutant forms of Pol IV co-purified under native conditions via epitope-tagged RDR2 (RDR2-FLAG). Total protein input and Anti-FLAG immunopurified protein were separated on an SDS 6% polyacrylamide gel and subjected to western blotting. NRPD2 and NRPD1-specific antibodies (validated in panel B), were used to successively detect corresponding Pol IV subunits in protein fractions co-purified with RDR2-FLAG (middle panels, right-hand lanes), then an anti-FLAG antibody was used to detect RDR2-FLAG (top panel, right-hand lanes). Staining with Coomassie solution was used to evaluate protein loading in the input fractions. (**E**) qRT-PCR performed on total RNA from inflorescences using random-primed cDNA synthesis and qPCR primers specific for the *AtSN1* retroelement. Following *ACT2* normalization, *AtSN1* transcript expression is plotted as the ratio of WT SucSul D4/sample. Error bars indicate the standard error of the mean for three technical replicates.

Native antibodies specific for the catalytic subunits of Pol IV (NRPD1 and NRPD2) were raised using a commercial service (Eurogentec). Two rabbits per target protein were inoculated, respectively, with a C-terminal peptide from NRPD1 (CLKNGTLESGGFSENP) or with an N-terminal peptide of NRPD/E2 (MPDMDIDVKDLEEFEC). Serum aliquots from the final bleeds were affinity purified on columns using the peptide corresponding to each original inoculation (Eurogentec). Antibody specificity was tested by extracting total protein from WT, *nrpd1-3* null and *nrpd/e2-2* null plants, then performing a western blot and observing the loss of the appropriately sized bands in each null mutant compared to WT samples (see Figure 1B). The monoclonal Anti-FLAG-HRP M2 antibody used to detect RDR2-FLAG was a commercial reagent (Sigma).

### Western blotting

Denaturing protein extraction was performed on inflorescence tissue of Arabidopsis following (52). Resulting protein pellets were resuspended in a buffer composed of 10% glycerol, 3% sodium dodecyl sulphate (SDS), 62.3 mM Tris–HCl pH 8.0, 1× Complete ethylenediaminetetraacetic acid (EDTA)-free Protease Inhibitor Cocktail (Sigma) and 2% β-mercaptoethanol. Protein amounts were quantified by Lowry (Bio-Rad), the concentrations were adjusted and 4× Laemmli Buffer (0.25 M Tris–HCl, 8% SDS, 40% glycerol, 0.01% bromophenol blue, 10% β-mercaptoethanol) was added before storage at −20°C. Samples were thawed at 95°C for 3 min. A total of 600 μg were separated on a 6% SDS-PAGE, transferred on a Immobilion-P membrane (Millipore IPVH00010). The membrane was blocked 30′, incubated overnight with the primary ‘anti-NRPD1’ antibody (1:5000 dilution), washed and incubated with the secondary antibody coupled to horseradish peroxidase. Chemiluminescent western signals were detected on film (Fuji Medical X-ray Medical Film) using the Lumi-Light Plus Western Blotting Substrate kit (Roche). The membrane was stripped for 12 min in Restore PLUS Western Blot Stripping Buffer (Thermo Scientific), washed, blocked and then incubated with the primary ‘anti-NRPD/E2’ antibody (1:2500 dilution).

### Protein co-immunopurification

About 170 mg of Arabidopsis inflorescences were ground in liquid nitrogen and then suspended in 1.5 ml lysis buffer (50 mM Tris–HCl pH 8.0, 150 mM NaCl, 5 mM MgCl_2_, 0,1% NP-40, 200 μl Protease inhibitor, 1 mM PMSF) with continued grinding at 4°C for 10 min. Resulting extracts were transferred to microfuge tubes and centrifuged at 16 000 rcf and 4°C for 5 min. An aliquot of each supernatant was stored at −20°C (input), then the remainder was transferred to a tube containing 50 μl of re-suspended Miltenyi (μMACS ‘DYKDDDDK’ kit, equivalent to anti-FLAG) beads and this mixture was incubated at 4°C for 35 min on a wheel (8 rpm). Miltenyi columns were installed in the manufacturer's magnetic stand, prepped with 200 μl of lysis buffer and progressively loaded with 200 μl volumes of the supernatant-bead mixtures. Each column was washed six times with lysis buffer. Remaining liquid was removed and 45 μl of preheated Laemmli buffer (95°C) was added for a 5 min incubation with the columns stoppered. Three additional 30 μl aliquots of Laemmli buffer (95°C) were added, allowing protein elution from the columns. The combined eluate was mixed, heated for 5 min at 95°C and stored at −20°C. For western blotting, 30 μl of input or 30 μl of each sample were used following the previous description. Monoclonal Anti-FLAG-HRP M2 (dilution 1:15 000) was used to detect RDR2-FLAG protein.

### Semi-quantitative/quantitative reverse transcription PCR (RT-PCR)

Total RNA was extracted from Arabidopsis inflorescence tissue following instructions of the RNeasy Kit (Qiagen), treated with DNase I (ThermoFisher Scientific) and then re-purified using phenol–chloroform extraction followed by ethanol precipitation. Then, 1 μg of DNase-treated RNA was aliquoted for random-primed cDNA synthesis using SuperScript IV Reverse Transcriptase (ThermoFisher Scientific) at 50°C. For each RT-PCR panel, a control omitting SuperScript IV was also prepared. For semi-quantitative RT-PCR fragments were amplified with *AtSN1* or *ACT2*-specific primers and visualized on an agarose gel after ethidium bromide staining. For quantitative RT-PCR, the synthesized cDNA was subjected to real-time PCR and detected via SYBR Green fluorescence in the LightCycler 480 II instrument (Roche Applied Science). See Supplementary Table S1 for primer sequences.

### Pol IV *in vitro* transcription assays

Transcription assays were carried out as described in (6). Briefly, 3 g of 3-week-old Arabidopsis seedlings were flash frozen in liquid-nitrogen and then lysed in 14 ml of lysis buffer (20 mM Tris–Cl, pH 7.6; 150 mM sodium sulfate; 5 mM magnesium sulfate; 20 μM zinc sulfate; 1 mM PMSF; 5 mM Dithiothreitol (DTT) and 1× Plant Protease Inhibitor Cocktail (Sigma)). Crude lysates were centrifuged at 18 000 rcf for 15 min and the soluble fractions were incubated with 25 μl of anti-FLAG M2 agarose resin (Sigma) for 2.5 h at 4°C to immunoprecipitate Pol IV-RDR2-FLAG complexes. The resin was washed twice with 15 ml of lysis buffer (without Plant Protease Inhibitor Cocktail) followed by 15 ml of low salt wash buffer (20 mM HEPES-KOH, pH 7.6; 100 mM potassium acetate; 5 mM magnesium sulfate; 20 μM zinc sulfate; 10% glycerol; 1 mM PMSF and 5 mM Dithiothreitol). The resin was then resuspended in low salt wash buffer to 50 μl, followed by addition of transcription buffer to a transcription reaction volume of 100 μl.

The template DNA, non-template DNA and RNA primer oligos were synthesized by Integrated DNA Technologies and polyacrylamide gel electrophoresis (PAGE)-purified. A total of 2 μM RNA primer was end-labeled using T4 polynucleotide kinase (NEB) in the presence of 25 μCi [γ-^32^P]-ATP (6000 Ci/mmol, Perkin Elmer) and in a total volume of 50 μl. Equimolar amounts of template DNA, end-labeled RNA primer and 10% excess of non-template DNA were mixed in the annealing buffer (30 mM HEPES-KOH pH 7.6, 100 mM potassium acetate), brought to 100°C and slowly cooled to room temperature to obtain the template used in the transcription assays.

Transcription reactions were carried out in a buffer with the following final composition: 20 mM HEPES-KOH pH 7.6, 100 mM potassium acetate, 60 mM ammonium sulfate, 10 mM magnesium sulfate, 10% v/v glycerol, 20 μM zinc sulfate, 0.1 mM PMSF, 1 mM DTT, 0.8U/μl Ribolock (Thermo Fisher), 1 mM each of ATP, GTP, CTP and UTP and 25 nM template. The reactions were incubated at room temperature for 1 h on a rotisserie, stopped by heat denaturation at 70°C for 5 min followed by desalting using Performa spin columns (Edgebio). Transcription reactions were adjusted to 0.3 M sodium acetate, and 15 μg of Glycoblue (ThermoFisher) and 3 volumes of isopropanol were added. Following centrifugation at 16 000 x g, 15 min, nucleic acid pellets were washed 2x with 70% ethanol, resuspended in 2x RNA loading dye (NEB) and resolved on 15% denaturing polyacrylamide gels (45x20 cm). The gels were dried for 2 h on a vacuum gel drier at 80°C and the signal was developed using autoradiography.

### Small RNA blot hybridization

Total RNA was extracted from Arabidopsis inflorescences using TRIzol reagent (Invitrogen) and size-fractionated as described in (53). Then, 9 μg low molecular weight RNA was resuspended in 8 μl RNA Loading Buffer (95% formamide, 0.025% bromophenol blue, 0.025% xylene cyanol FF, 5 mM EDTA, 0.025% SDS, pH 8.5). Samples were heated to 95°C for 3 min and separated on an 16% polyacrylamide gel. RNA loading was documented using Ethidium bromide gel staining followed by UV transillumination. Size-separated RNAs were transferred to a nylon membrane (Hybond-N+, GE Healthcare) by electroblotting and UV cross-linked (140 mJ/cm^2^). Different ^32^P 5′-end-labeled DNA oligonucleotides were used for successive hybridizations in PerfectHyb Plus Buffer (Sigma) overnight at 35–40°C, depending on the probe. The membrane was washed three times for 20 min in Wash Buffer (0.3 M NaCl, 30 mM sodium acetate, 0.5% SDS, pH 7.0), exposed to a phosphor-imager screen for 3 days, then the screen was scanned using a Typhoon Multimode-imager (GE Healthcare). Each probe was stripped with boiling 0.1% SDS (two times, 20 min) prior to the next hybridization (see Supplementary Table S1 for probe sequences).

### Small RNA sequencing

Total RNA was extracted from Arabidopsis inflorescences using TRIzol reagent, treated with DNase I (ThermoFisher Scientific) and then re-purified using phenol-chloroform extraction followed by ethanol precipitation. About 5 μg of DNase-treated RNA was sent for library preparation: ∼15–94 nt RNAs were selected by polyacrylamide size-separation, Illumina TruSeq small RNA-seq libraries were prepared (2× replicates per genotype) and the libraries sequenced on an HiSeq 2500 platform (1 × 125 bp, Fasteris SA, http://www.fasteris.com). The 3′-adapter sequences were removed by the Fasteris data pipeline. These trimmed reads were quality-filtered (*q* > 30) using Cutadapt v1.14 (https://doi.org/10.14806/ej.17.1.200) and mapped to the Arabidopsis TAIR10 reference genome using Bowtie v1.2.2 (http://bowtie-bio.sourceforge.net/index.shtml) without allowing mismatches but permitting multi-mapped reads (Supplementary Tables S7 and 8). Small RNA counts were extracted using ShortStack v3.8.5 (http://sites.psu.edu/axtell/software/shortstack/) and normalized by the total number of mapped reads. Boxplots of 24 nt siRNAs were generated in R, counting reads per kb per million mapped (RPKM) within *pol IV* differentially methylated regions (DMRs) (loci hypomethylated in *nrpd1-51* versus WT SucSul). Wilcoxon rank sum tests were then performed using the ggpubr package (Supplementary Table S12).

### DNA methylation detection

DNA was extracted from Arabidopsis inflorescences using the Nucleon Phytopure kit (GE Healthcare) following the manufacturer's recommendations and including RNase A treatment. *Chop-PCR:* 90 ng of genomic DNA was digested with HaeIII or AluI, alongside reactions aliquots from which the restriction enzyme was omitted (no digest controls), as described in (21). Target loci were then amplified by PCR or qPCR with primers flanking the restriction sites (Supplementary Table S1). *Amplicon-based bisulfite sequencing* was performed using the EpiMark Bisulfite Conversion Kit (New England Biolabs). PCR fragments amplified using bisulfite-treated DNA and the primers AtSN1-Bi-F and AtSN1-Bi-R (Supplementary Table S1) were cloned into pGEM-T-Easy and Sanger sequenced. For each genotype, at least 38 *AtSN1* bisulfite clones were aligned in Geneious, analyzed using CyMATE (http://www.cymate.org/) and plotted in Excel and R. Boxplots of percentage DNA methylation on three distinct *AtSN1* intervals were generated, and Wilcoxon rank sum tests were performed using the ggpubr package in R. *Whole-genome bisulfite sequencing* (WGBS) was performed by Beijing Genomics Institute (BGI, https://www.bgi.com/) on 18 samples (2× replicates, 9 genotypes) using a 2 × 150 bp Illumina HiSeq run to obtain ∼38 million reads per sample. WGBS read quality and mapping stats are provided in Supplementary Table S4.

### Differentially methylated regions

Adapter and quality trimming (*q* > 20) were performed on the WGBS data using TrimGalore (v0.4.4). Clean reads were mapped to the Arabidopsis reference genome (TAIR10) using Bismark v0.18.1 (https://www.bioinformatics.babraham.ac.uk/projects/bismark/). Methylation information for each methylcytosine context (CG, CHG and CHH) was extracted (*bismark_methylation_extractor*) after de-duplication (*deduplicate_bismark*). DMRs were identified from the Bismark analysis files using the BSseq R package (v1.10.0). WT SucSul was used as the reference sample for calling DMRs. These DMRs were called using the BSseq default t-stats quantile cutoff and only including cytosine positions supported by at least four reads in both replicates (Supplementary Tables S5 and 6). Furthermore, the DMRs were filtered using the following stringent criteria: minimum 100 bp length, more than five total Cs and minimum differences in methylation level of 40, 20 and 10%, respectively, for the CG, CHG and CHH sequence contexts. All graphics were generated in R using ggplot2 and ggpubr packages (https://www.r-project.org/).

### Protein sequence alignment

Amino acid (aa) sequences for the largest subunits of DNA-dependent RNA polymerases were obtained from Uniprot, NCBI and Phytozome (Supplementary Table S10), including NRPD1 (Pol IV) from 17 diverse species (54). All sequences were imported into Geneious (v11.1.5) (https://www.geneious.com). Sc.RPB1 was hand-annotated with evolutionarily conserved ‘Domains A to H’ of Pol I/II/III/IV/V and with point mutations known to affect Pol II activity (46,48,55–57). Ath.NRPD1 was annotated with the *nrpd1-47, nrpd1-49* and *nrpd1-50* mutations (Supplementary Figure S1B). Ath.NRPD1 and Ath.NRPE1 were annotated with the DeCL/DUF3223 domains and the WG repeat region (44,46,58). NRPD1 sequences were aligned using MUSCLE (v3.8.425, default parameters), then Ath.NRPB1, Sc.RPB1 and Ath.NRPE1 were introduced to this alignment using the Geneious profile-based aligner. Figure 4, and Supplementary Figures S2.A and S4 represent views of the same global alignment with species shifted top/bottom, or omitted depending on space available in each panel (e.g. a long insertion between ‘Domain A’ and ‘Domain B’ of *Ginkgo biloba* NRPD1 and *P. canariensis* NRPD1 prevented their inclusion in Figure 4). We generated a Hidden Markov Model based on the 22 aa Pol IV-specific motif region in the alignment and queried UniProt Reference Proteomes using hmmsearch (https://www.ebi.ac.uk/Tools/hmmer/). The list of high quality hits (*E*-value < 0.01) included proteins from 46 distinct plant species: all proteins >1300 aa were downloaded and re-analyzed by the same procedures as outlined above to scan for NRPB1, NRPD1 and NRPE1 domains/subdomains (Supplementary Table S11).

## RESULTS

### Point mutations in the N-terminus of Pol IV’s largest subunit

To obtain an allelic series of *nrpd1* mutations in Arabidopsis, EMS-mutagenized seed pools were screened using a *SUC2::IR-SUL* transgenic reporter. In this system (43), the Arabidopsis *SUL* mRNA is silenced by *SUL* dsRNA arising from an inverted-repeat (*IR-SUL*) under control of the Arabidopsis *SUC2* promoter (Supplementary Figure S1A). Plants carrying the silencer (WT SucSul) show vascular bleaching due to knock-down of the SUL magnesium chelatase, in contrast to uniformly green leaves of untransformed plants (WT Col-0) (Figure 1A). To preclude recovery of *dcl4* mutations, which are frequently retrieved in *SUC2::IR-SUL* screens (59), the mutagenized parental line also harbored a DCL4 transgene driven by the CaMV 35S promoter (WT SucSul D4). The subsequent M2 plant generation was screened for individuals that lack vascular bleaching despite the presence of *SUC2::IR-SUL*; these candidates were analyzed for *NRPD1* gene mutations by PCR amplification and Sanger sequencing (Supplementary Figure S1B).

The five point mutations isolated in *NRPD1* were designated *nrpd1-47* to *nrpd1-51*, numbering up from the last reported *nrpd1* alleles (42). The *nrpd1-48* mutation generates stop codons in Exon 3 due to a splicing defect, whereas the *nrpd1-51* mutation substitutes an early stop for tryptophan (W664*), suggesting that both are *pol IV* null alleles. By contrast, *nrpd1-47, nrpd1-49* and *nrpd1-50* are missense mutations in the NRPD1 N-terminus within and adjacent to the evolutionarily conserved ‘Domain A’ of multisubunit RNA polymerases (57). The corresponding WT NRPD1 amino acids are not thought to contribute to the Pol IV active site (Figure 1A) (9,48). As expected, given the premature stop codons, NRPD1 protein was not detectable in *nrpd1-48* or *nrpd1-51* point mutants, phenocopying the *nrpd1-3* null mutant (T-DNA insertion). However, nrpd1-47, *nrpd1-49* and *nrpd1-50* missense mutants expressed WT or slightly higher levels of NRPD1 protein (Figure 1B, top panel). The second largest subunit of Pol IV, NRPD2, accumulated equally in WT controls and in all the *nrpd1* mutants, but was not detectable in the *nrpd/e2-2* null mutant control (Figure 1B, middle panel).

### Genetic lesions in conserved ‘Domain A’ of NRPD1

To explore the impact of NRPD1 N-terminus mutations on Pol IV, we aligned protein sequences of RPB1 from yeast (*Saccharomyces cerevisiae*, Pol II), NRPB1 from Arabidopsis (Pol II), NRPD1 from 17 phylogenetically diverse plant species and NRPE1 from Arabidopsis (Pol V). Focusing on conserved ‘Domain A’ in this alignment (Supplementary Figure S2A), we noticed that *nrpd1-47* and *nrpd1-49* affect amino acids corresponding to a zinc-binding domain of yeast Pol II (56,57). The *nrpd1-50* mutation does not change a residue in ‘Domain A’ so we address its context in a later section. Comparison of the Pol IV amino acids mutated in *nrpd1-47* and *nrpd1-49* to homologous positions in yeast Pol II situates these highly conserved residues near the RNA exit channel of the Pol II quaternary structure. This inference led us to hypothesize that the *nrpd1-47* and *nrpd1-49* missense mutations could disrupt a putatively similar zinc-binding domain in Pol IV, perhaps interfering with Pol IV-RDR2 assembly (Figure 1C).

### Pol IV-RDR2 assembly in NRPD1 N-terminus missense mutants

To test whether NRPD1 carrying N-terminal mutations can assemble with other Pol IV subunits and RDR2, we generated plants expressing FLAG-epitope tagged RDR2 that rescues the *rdr2-2* null mutant (Supplementary Figure S2B). Crossing this *RDR2-FLAG* line to *nrpd1-47*, nrpd1-49, *nrpd1-50* and *nrpd1-51* mutants, respectively, we selected F2 progeny in which each *nrpd1* mutation was homozygous and the RDR2-FLAG protein was expressed. Anti-FLAG beads were used to immunopurify RDR2-FLAG from protein extracts obtained from the *RDR2-FLAG* WT *NRPD1* or *RDR2-FLAG nrpd1* mutant plants. After SDS-PAGE and western blotting, RDR2-FLAG was detected in the WT *NRPD1* background and in all the *nrpd1* mutants (Figure 1D, α-FLAG IP lanes, top panel). An NRPD1-specific antibody detected the largest subunit of Pol IV in all samples except the *nrpd1-51* null mutant. Likewise, an NRPD2-specific antibody detected the second largest subunit of Pol IV in all samples except *nrpd1-51* (Figure 1D, α-FLAG IP lanes, middle panels). Based on these data, we conclude that the NRPD1 N-terminus residues mutated in *nrpd1-47, nrpd1-49* and *nrpd1-50* are not individually essential for Pol IV-RDR2 assembly.

### N-terminal *nrpd1* missense mutations disrupt *AtSN1* retroelement silencing

To assess how distinct *nrpd1* mutations affect Pol IV function in TE silencing, we measured the derepression of a known Pol IV target, *AtSN1*, by qRT-PCR (Figure 1E). In WT plants *AtSN1* is silenced and transcripts do not accumulate, but transcripts are detected in *nrpd1* null mutants (44). The NRPD1 N-terminus missense mutants (nrpd1-47, *nrpd1-49, nrpd1-50*) showed less *AtSN1* transcript accumulation than null mutants (*nrpd1-48, nrpd1-51, nrpd1-3*) (Figure 1E). This suggests that *AtSN1* is still partially silenced by Pol IV in plants expressing N-terminally-mutated NRPD1, in contrast to full *AtSN1* derepression in null mutants (Figure 1E, Supplementary Figure S2C). Accordingly, cytosine methylation assayed at *Hae* III sites in *AtSN1* was only partially lost in *nrpd1-47, nrpd1-49* and nrpd1-50, but more severely reduced in *nrpd1-48, nrpd1-51* and nrpd1-3 (Supplementary Figure S2D).

### Pol IV *in vitro* transcription assays

A previously reported C67S mutation in yeast RPB1 (*rpo21-27*) causes growth defects (56) and corresponds to the NRPD1 position mutated in the Arabidopsis *nrpd1-47* (C56Y) mutant. Another such yeast mutation, *rpo21-30* (H80Y), affects a position adjacent to the NRPD1 amino acid mutated in *nrpd1-49* (Supplementary Figure S2A). These *rpo21-27* and *rpo21-30* mutations both reduce the transcriptional activity of Pol II in yeast (56). Therefore, an alternative hypothesis to Pol IV-RDR2 assembly being disrupted in *nrpd1-47* or *nrpd1-49* would be that Pol IV transcriptional activity is reduced by these genetic lesions.

**Figure 2.**
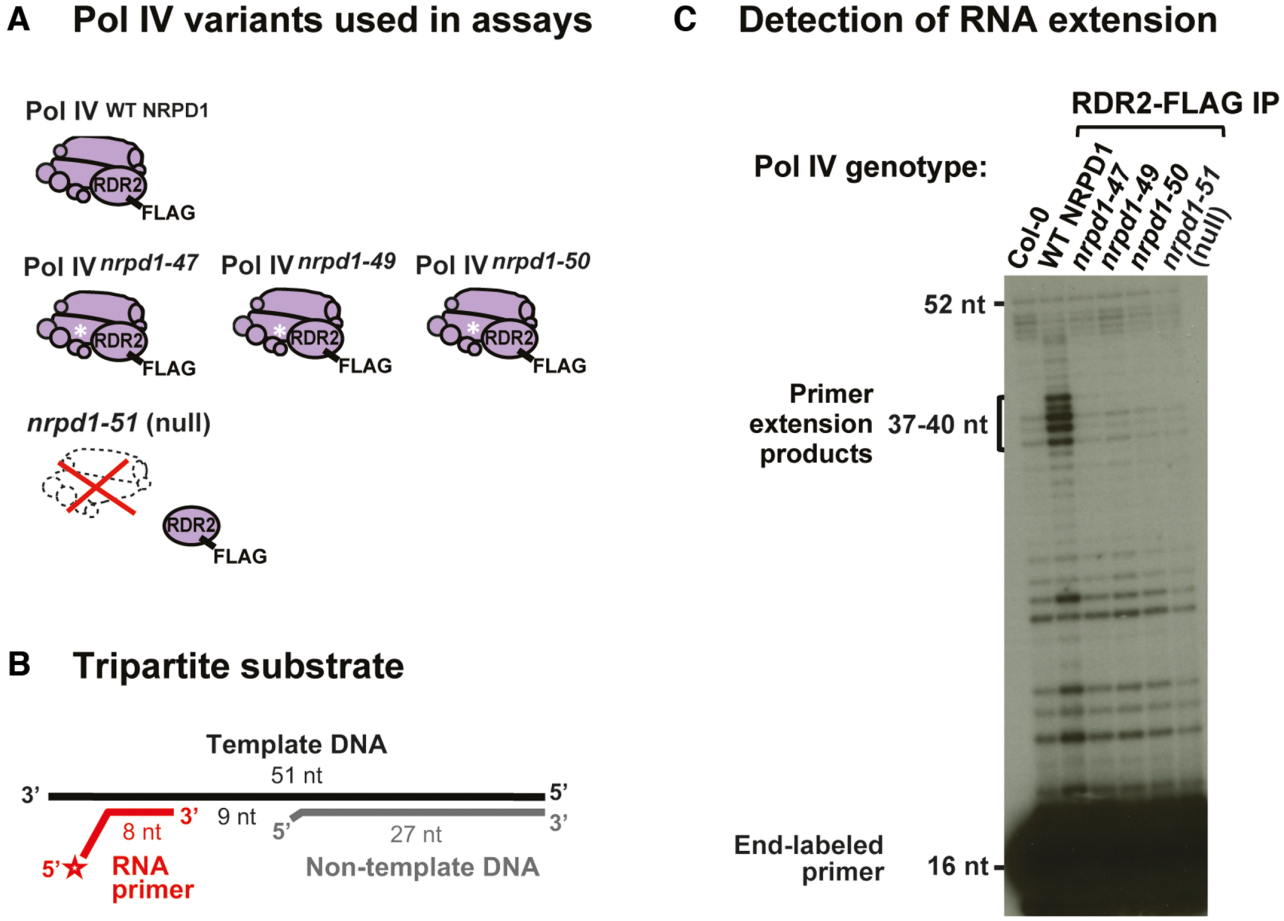
Pol IV *in vitro* transcription assays. (**A**) The Pol IV complexes assembled with WT NRPD1 or with *nrpd1-47, nrpd1-49* or *nrpd1-50* mutant variants (Pol IV^WT NRPD1^, Pol IV*^nrpd1-47^*, Pol IV*^nrpd1-49^*or Pol IV*^nrpd1-50^*) were co-purified with RDR2-FLAG and then used for *in vitro* transcription assays. *nrpd1-51* is a null mutation in Pol IV, abolishing its co-purification with RDR2-FLAG and thus serves as a negative control for Pol IV activity. (**B**) The tripartite substrate used for the assays is composed of a template DNA, a non-template DNA and a radiolabeled RNA primer. (**C**) Transcription assays for each Pol IV variant (Pol IV^WT NRPD1^, Pol IV*^nrpd1-47^*, Pol IV*^nrpd1-49^*, Pol IV*^nrpd1-50^*or the null mutant *nrpd1-51*). An assay using protein from non-transgenic plants (Col-0) provides an additional negative control. The 5′-end radiolabeled, 16 nt RNA primer is seen at the gel bottom and *in vitro* RNA extension products are visible in the 37–40 nt range near the top third of the gel.

To test whether Pol IV enzymatic activity is affected by NRPD1 N-terminus mutations, we performed *in vitro* transcription assays following an established protocol (6,9). Precipitation of RDR2-FLAG using Anti-FLAG beads allowed co-purification of Pol IV^WT NRPD1^, Pol IV*^nrpd1-47^*, Pol IV*^nrpd1-49^* or Pol IV*^nrpd1-50^* using the respective WT or *nrpd1* mutant backgrounds (Figure 2A). *nrpd1-51* is the *pol IV* null mutant in which Pol IV-RDR2 assembly is not possible (Figure 1D). The assays utilize a 51 nt DNA template oligonucleotide annealed for 27 bp with a non-template DNA strand and hybridized for 8 bp with an end-labeled RNA oligonucleotide primer (Figure 2B), thus mimicking a transcription elongation complex. Pol IV can elongate the RNA primer in a DNA templated fashion, but terminates 12-16 nt after encountering the base-paired non-template DNA strand (6), generating 37-40 nt transcripts, as observed for Pol IV assembled using WT NRPD1 (Figure 2C). By contrast, Pol IV co-purified with RDR2-FLAG in the *nrpd1-47, nrpd1-49* or *nrpd1-50* backgrounds, respectively, showed little activity, resembling the *nrpd1-51* null mutant or Col-0 negative control (Figure 2C). We conclude that the Pol IV *in vitro* activity is crippled or abolished by the mutations in the NRPD1 N-terminus.

### siRNA biogenesis and DNA methylation in NRPD1 N-terminus mutants

**Figure 3.**
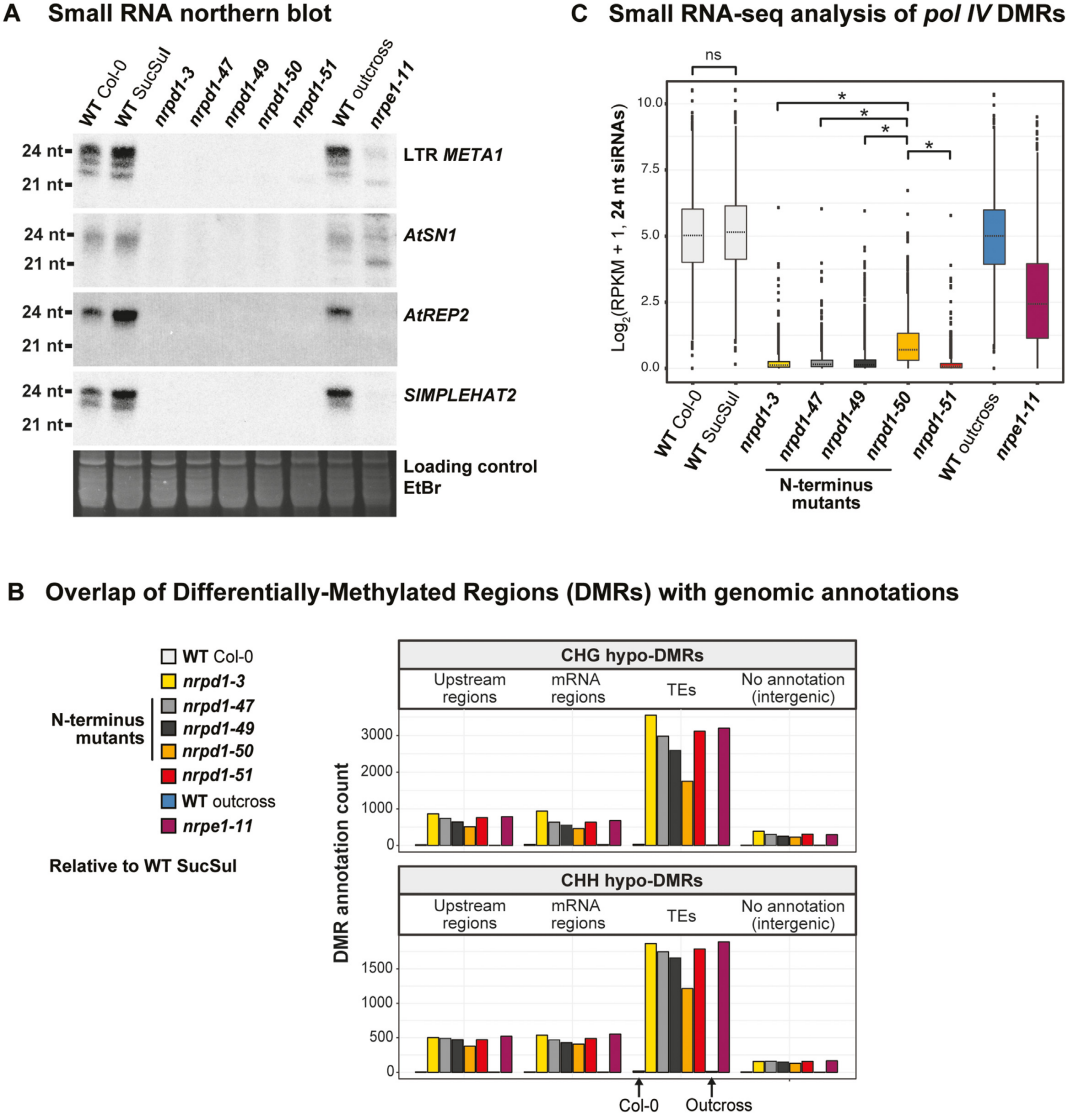
N-terminal mutations in NRPD1 nearly abolish 24 nt siRNA accumulation and RdDM. (**A**) Small RNA northern blot using several probes at Pol IV-RdDM targets: LTR *META1, AtSN1, AtREP2, SIMPLEHAT2*. Equal loading was tested by imaging the gel stained with ethidium bromide (EtBr) prior to blotting. (**B**) Overlap of Differentially-Methylated Regions (DMRs) with genomic annotations. DMRs were identified in each sample relative to WT SucSul using whole-genome bisulfite sequencing (2× replicates per sample genotype). Regions with less methylation than WT SucSul (hypo-DMRs) were counted upstream of genes, in mRNA regions, in TEs and in regions lacking annotation (intergenic regions). CHG sequence and CHH sequence context DMRs are tabulated separately (above and below, respectively). (**C**) Small RNA-seq analysis of *pol IV* DMRs. Small RNA-seq was performed on the same genotypes as analyzed in panel B. After mapping, 24 nt siRNAs were counted in putative Pol IV-RdDM regions (i.e. *nrpd1-51* hypo-DMRs; see Supplementary Tables S5 and S9). Pairwise statistical comparisons were performed using the Wilcoxon rank sum test: *P*-values < 0.01 were treated as not significant (ns), whereas *P*-values ≥ 0.01 were treated as significant (*). Only comparisons between WT Col-0 and WT SucSul controls, as well as comparisons between *nrpd1-50* and the other *nrpd1* mutant alleles are shown explicitly with brackets in Figure 3.

Defects in Pol IV transcription would limit production of 24 nt siRNAs *in vivo* (8,46,48), so we used RNA blot hybridization to test whether siRNA levels changed in the *nrpd1* N-terminus mutants. A probe for the LTRs of *META1 Copia* retrotransposons detected 24 nt siRNAs in WT Col-0 and WT SucSul (Figure 3A). These siRNAs were not detected in *nrpd1-3* or *nrpd1-51* null mutants, but they were also not observed in any N-terminus missense line (Figure 3A). Similar results were obtained for siRNAs from the *AtSN1* retroelement, as well as from the *AtREP2* and *SIMPLEHAT2* DNA transposons. Outcrossing nrpd1-51 to WT Col-0 followed by selection of homozygous WT *NRPD1* in the F2 generation restored siRNA levels (Figure 3A).

The apparent loss of siRNAs (and undetectable Pol IV activity *in vitro*) in *nrpd1-47, nrpd1-49* and *nrpd1-50* missense lines was surprising because these mutants only showed a partial loss of *AtSN1* silencing *in vivo*. We thus pursued genome-scale analyses to chart the global impact of NRPD1 N-terminus mutations on RdDM. Whole-genome bisulfite sequencing (WGBS) was performed on DNA from WT Col-0 and SucSul controls, *nrpd1* mutants, *nrpd1-51* outcrossed to Col-0 and the *nrpe1-11* (*pol V*) null mutant. WGBS reads were mapped to the Arabidopsis genome and Differentially-Methylated Regions (DMRs) were called relative to WT SucSul (2x replicates per sample, Supplementary Table S5). Comparison of WT Col-0 to WT SucSul revealed only 100 regions with reduced methylation (hypo-DMRs), indicating that both controls display similar patterns of DNA methylation. In the *nrpd1-3* null mutant, 3553 TE regions, 863 regions upstream of genes and 935 mRNA regions were detected overlapping CHG hypo-DMRs (relative to WT SucSul); in addition, 1866 TEs, 502 upstream regions and 538 mRNA regions were found overlapping CHH hypo-DMRs in the *nrpd1-3* mutant (Figure 3B, yellow bars). *nrpd1-51* showed frequencies of hypo-DMRs comparable to *nrpd1-3* (Figure 3B, red bars). Slightly fewer hypo-DMRs were detected in *nrpd1-47* and *nrpd1-49* N-terminus mutants (Figure 3B, dark grey bars), although these mutations nearly phenocopied *pol IV* null alleles in the CHH methylation context. By contrast, the *nrpd1-50* N-terminus mutant displayed far fewer hypo-DMRs than either null allele, an effect most apparent in TEs (Figure 3B, orange bars). Pol IV-dependent DNA methylation was globally resettable, with only 57 total hypo-DMRs recovered after *nrpd1-51* outcross (Figure 3B, blue bars). Finally, the number of hypo-DMRs in the *pol V* null mutant (Figure 3B, purple bars) was similar to *pol IV* null alleles (*nrpd1-3* and *nrpd1-51*) and larger than any of the *nrpd1* N-terminus missense alleles.

Although 24 nt siRNAs could not be detected by northern blot in *nrpd1-50* plants (Figure 3A), the fewer DMRs in *nrpd1-50* compared to *pol IV* null plants suggested that Pol IV-dependent DNA methylation continued at hundreds of chromosomal targets in *nrpd1-50*. To better understand the role of the NRPD1 N-terminus in Pol IV function, we performed small RNA-seq on the same samples as were analyzed by WGBS. The abundance of 24 nt siRNAs was quantified at all regions of Pol IV-dependent DNA methylation (Figure 3C) (2x replicates per sample, Supplementary Table S9). Boxplots for WT Col-0 and SucSul controls were indistinguishable, with median values near 35 reads per kilobase per million reads mapped (RPKM), whereas medians for *nrpd1-3* and *nrpd1-51* null mutants were drastically reduced (Figure 3C, note log2 scale). Similar to the DMR results above, Pol IV-dependent siRNA production was restored after the *nrpd1-51* null mutation was outcrossed (Figure 3C, blue boxplot). However, 24 nt siRNA levels in the nrpd1-50 N-terminus mutant (Figure 3C, Supplementary Figure S3) were significantly higher than those of *pol IV* null mutants. This *nrpd1-50* median represented trace amounts of 24 nt siRNAs, ∼55 times less than WT SucSul, explaining why the less sensitive RNA blot technique detected no 24 nt siRNAs (Figure 3A).

In summary, NRPD1 N-terminus mutations impair 24 nt siRNA accumulation and DNA methylation with varying degrees of potency. The strongest alleles, *nrpd1-47* and *nrpd1-49*, disrupted a putative zinc-binding domain in NRPD1 ‘Domain A’ that is conserved in the largest subunits of all multisubunit RNA polymerases (46,56). Thus, both *nrpd1-47* and *nrpd1-49* nearly phenocopied the siRNA and DNA methylation defects of *pol IV* null mutants. By contrast, the *nrpd1-50* mutation adjacent to ‘Domain A’ (Figure 1A, Supplementary Figure S4) caused less severe deficiencies, preserving trace 24 nt siRNA levels and residual DNA methylation at certain Pol IV-RdDM targets.

### A signature Pol IV motif uniquely conserved in NRPD1

**Figure 4.**
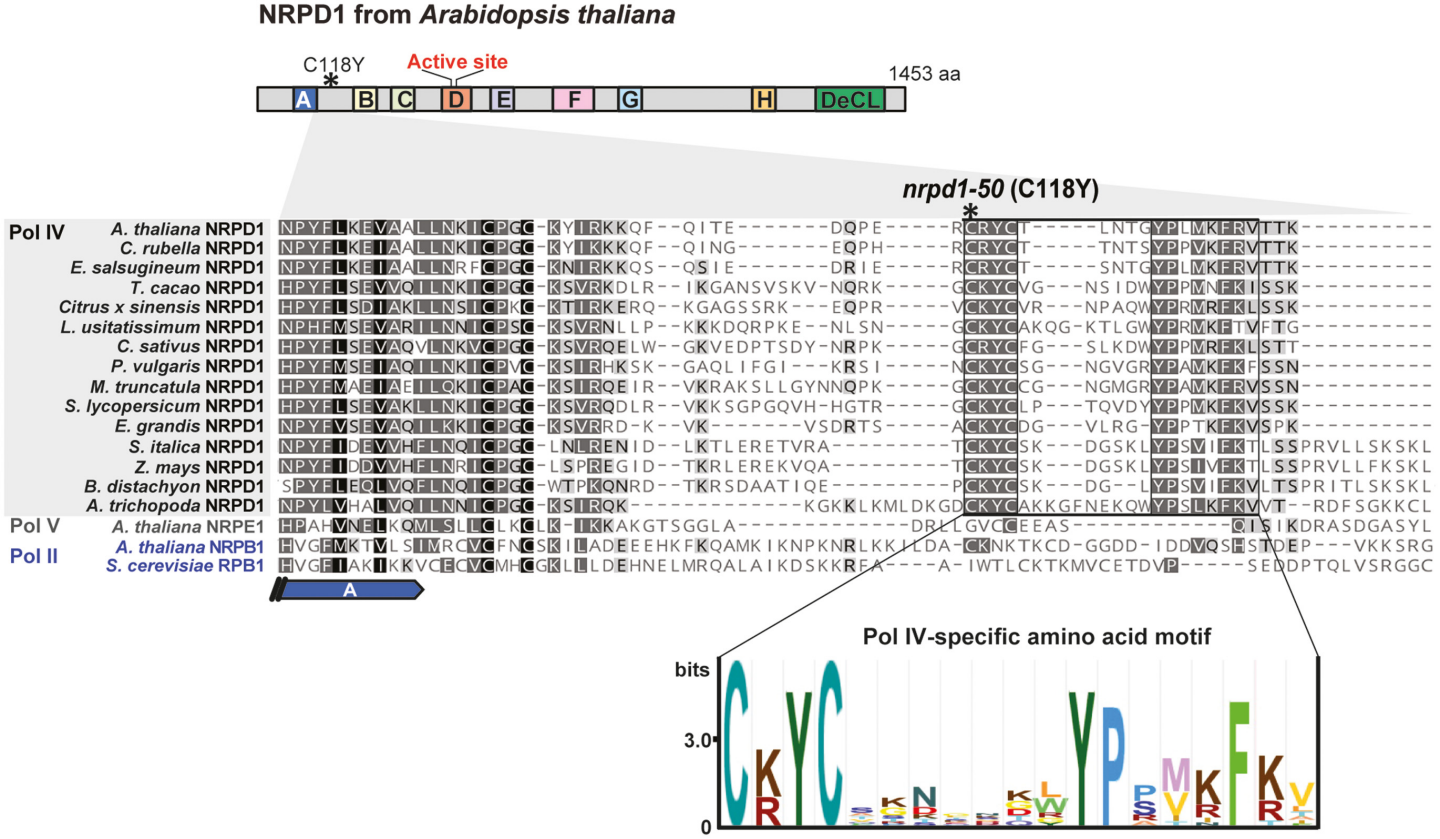
Mutation in NRPD1 adjacent to conserved ‘Domain A’ reveals a Pol IV-specific motif. Above: Diagram showing the position of the *nrpd1-50* mutation (C118Y) in NRPD1, the largest subunit of Pol IV, relative to this subunit's Domains A to H (conserved in the largest subunits of all nuclear RNA polymerases), and its C-terminal ‘Defective in Chloroplasts and Leaves’ (DeCL) domain. Middle: Alignment of amino acids adjacent to ‘Domain A’ in NRPD1 from the species *Arabidopsis thaliana, Capsella rubella, Eutrema salsugineum, Theobroma cacao, Citrus* x *sinensis, Linum usitatissimum, Crocus sativus, Phaseolus vulgaris, Medicago truncatula, Eucalyptus grandis, Solanum lycopersicum, Setaria italica, Zea mays, Brachypodium distachyon* and *Amborella trichopoda*. Included for comparison are NRPE1, the largest subunit of Pol V from Arabidopsis; NRPB1, the largest subunit of Pol II from Arabidopsis; and RPB1, the largest subunit of Pol II from *Saccharomyces cerevisiae*. Below: The alignment reveals a Pol IV-specific protein motif starting at Arabidopsis NRPD1 amino acid 118, which is composed of a C[KR]YC box followed by a 5–10 amino acid (aa) spacer and then by a YPx[MV][KR]F[KR] box.

Inspecting the *nrpd1-50* mutation (C118Y) in our NRPD1 alignment, we discovered an evolutionarily conserved protein motif composed of a C[KR]YC box followed by a 5–10 amino acid (aa) spacer and then by a YPx[MV][KR]F[KR] box (Figure 4). We initially found this motif in NRPD1 of 17 species ranging from the basal angiosperm *Amborella trichopoda* to the conifer *Pinus canariensis* (Figure 4, Supplementary Figure S4; Supplementary Table S10). However, it was absent in the corresponding regions of Arabidopsis NRPB1 (Pol II), Arabidopsis NRPE1 (Pol V) and yeast RPB1 (*S. cerevisiae*, Pol II). To identify more proteins with this motif, we generated a hidden Markov model based only on the motif region in our NRPD1 alignment and queried UniProt Reference Proteomes using hmmsearch (https://www.ebi.ac.uk/Tools/hmmer/). High-confidence matches to RNA polymerase subunits (*E*-value < 0.01, length > 1300 aa) included proteins from 46 plant species. These proteins contained close matches to Domains ‘A through H’ and the ‘Defective in Chloroplasts and Leaves’ domain (DeCL), as expected for NRPD1 or NRPE1 but not for NRPB1. Moreover, all identified subunits lacked the C-terminal extension containing WG/GW motifs, which is important for NRPE1 function in Pol V (54,58,60) (Supplementary Figure S4 and Table S11). We conclude that the amino acid position mutated in *nrpd1-50* is part of a signature Pol IV motif not found in Pol II, Pol V or other eukaryotic RNA polymerases.

### The Pol IV-specific motif safeguards robust TE methylation patterning

**Figure 5.**
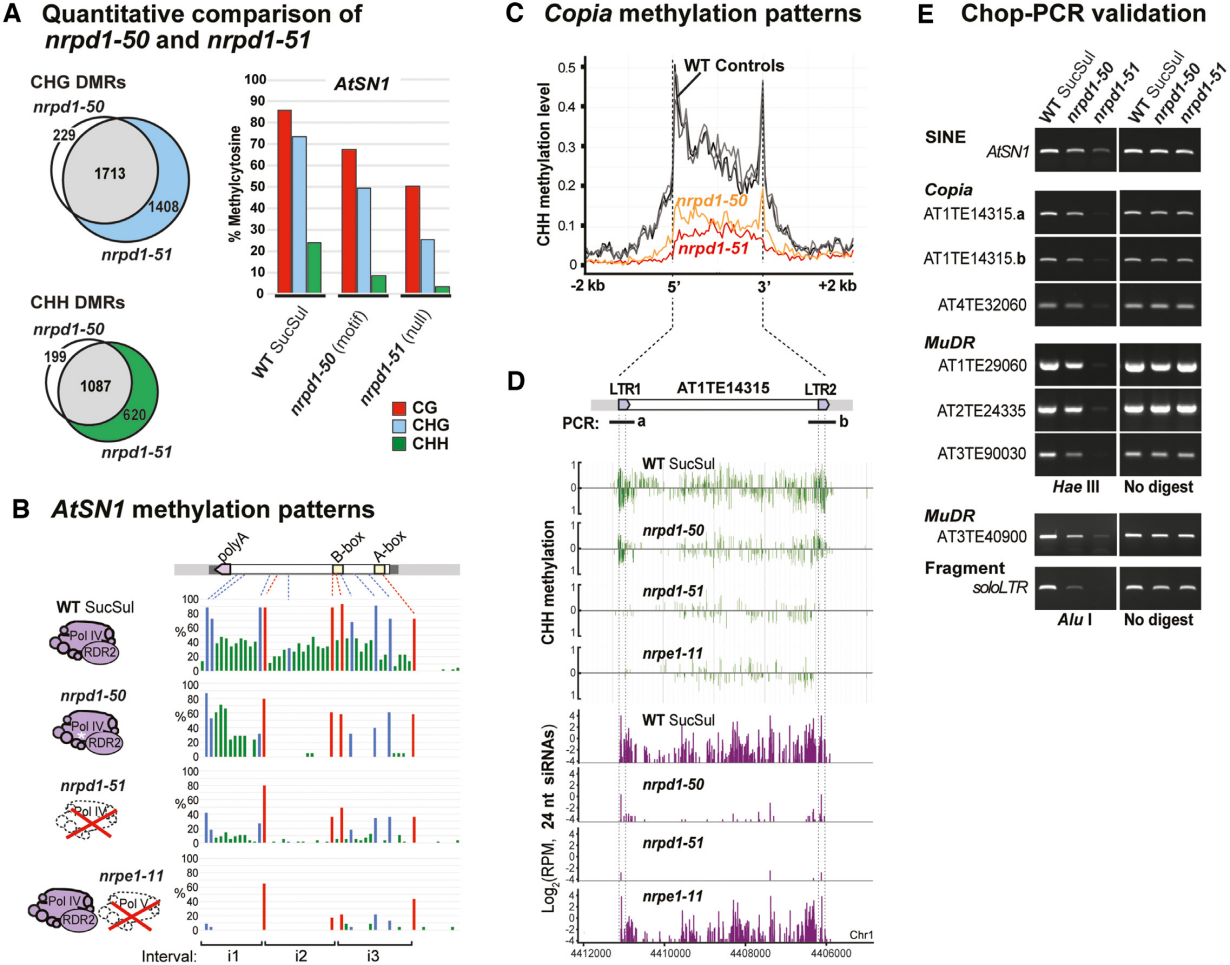
The Pol IV-specific motif in NRPD1 is critical for robust TE methylation patterning. (**A**) Left: Venn diagram of DMRs for CHG and CHH methylation contexts in *nrpd1-50* and *nrpd1-51* mutants. Right: total percentage of methylcytosine at the *AtSN1* locus in the three cytosine sequence contexts (CG, CHG and CHH) for WT SucSul, *nrpd1-50* and *nrpd1-51* mutants (*n* ≥ 38 clones/sample). (**B**) *AtSN1* methylation patterns: per site CG, CHG and CHH methylation, respectively, are shown as red, blue and green vertical lines. Wilcoxon rank sum tests were performed to compare WT SucSul, nrpd1-50 and *nrpd1-51* methylation within three different intervals of the *AtSN1* region (i1, i2 and i3; see Supplementary Figure S5A). (**C**) *Copia* methylation patterns: metaplot of CHH methylation levels on *Copia* elements between the annotated 5′ and 3′-ends, with 2 kb of genomic context included upstream and downstream. (**D**) Detail of CHH methylation (green vertical lines) and 24 nt siRNAs (purple vertical lines) for the *Copia* retrotransposon AT1TE14315 in WT SucSul, *nrpd1-50, nrpd1-51* and *nrpe1-11* (*pol V* null). Long terminal repeats LTR1 and LTR2 are nearly identical subfeatures of AT1TE14315 that differ at five genomic positions and each contain one *Hae* III site. (**E**) Chop-PCR: genomic DNA from WT SucSul, *nrpd1-50* and *nrpd1-51* was digested with methylation-sensitive restriction enzymes (rows 1–7 with *Hae* III; rows 8 and 9 with *Alu* I) followed by PCR. Successful PCR indicated that the template DNA was methylated (protected), whereas less amplification indicated less DNA methylation. Loci tested were: the SINE element *AtSN1*; the *Copia* elements AT1TE14315 (a and b correspond to LTR1 and LTR2, panel D) and AT4TE32060; the *MuDR* elements AT1TE29060, AT2TE24335, AT3TE90030 and AT3TE40900; and the *soloLTR* fragment. ‘No digest’ controls are PCR products from assays omitting the restriction enzyme.

Comparison of the hypo-DMRs common to *nrpd1-50* missense and *nrpd1-51* null mutants showed that the Pol IV-specific motif was critical for CHG methylation at 1942 loci and for CHH methylation at 1286 loci. An additional 1408 CHG and 620 CHH hypo-DMRs were detected only in the *nrpd1-51* null plants, in which Pol IV does not assemble (Figure 5A, pie charts). We used amplicon-based bisulfite sequencing to precisely quantify DNA methylation changes across the *AtSN1* retrotransposon locus. In the WT control 86% of CG sites, 73% of CHG sites and 24% of CHH sites were methylated (Figure 5A, bar chart). All three cytosine contexts in *AtSN1* showed less methylation in nrpd1-50, but these levels were reduced much further in nrpd1-51 null plants. Pol IV-RdDM, scored as CHH methylation, was distributed evenly across *AtSN1* in WT SucSul and was uniformly lost in *nrpd1-51* (Figure 5B). By contrast, the *nrpd1-50* methylation pattern displayed a striking discontinuity: CHH methylation was erased at the *AtSN1* 5′-end, over A and B-box promoter elements, but it remained intact near the 3′ polyA tract. Wilcoxon rank sum analysis of WT versus *nrpd1-50* amplicons supports the assessment that CHH methylation remained within the 3′ *AtSN1* interval (i1) in the mutant, unlike at upstream *AtSN1* subfeatures (i2, i3; Figure 5B and Supplementary Figure S5A). This trend was also noted in our WGBS data (Supplementary Figure S5B). Thus, residual *AtSN1* methylation and partial *AtSN1* silencing (see Figure 1E) support the hypothesis that the *nrpd1-50* mutant expresses a partially functional Pol IV*^nrpd1-50^* enzyme.

To further explore patterns of residual methylation in *nrpd1-50*, we surveyed other TE annotations in our WGBS data. Metaplots of CHH methylation across LTR/*Copia, MuDR* and *Helitron* elements revealed symmetrical peaks at the TE extremities in WT plants (Figure 5C and Supplementary Figure S5). These peaks remained sharp in *nrpd1-50*, whereas the overall methylation profile flattened in *nrpd1-51* null plants. Notably, CHH methylation at *Copia* long-terminal repeats (LTRs) was less sensitive to nrpd1-50 than to *nrpd1-51*, whereas TE body methylation was reduced in *nrpd1-50* and *nrpd1-51* (Figure 5C and Supplementary Figure S5). Similar effects were evident at individual *Copia* elements: AT1TE14315 body methylation was reduced in both *nrpd1-50* and *nrpd1-51* mutants, whereas LTR methylation peaks remained intact in *nrpd1-50* (Figure 5D, green tracks). This residual LTR methylation correlated with trace 24 nt siRNA accumulation in *nrpd1-50*; by contrast, the *pol V* mutant (*nrpe1-11*) erased CHH methylation with only a slight loss in siRNAs (Figure 5D, purple tracks). Numerous TEs had profiles similar to Figure 5D, with swaths of DNA methylation lost in *nrpd1-51* but remaining partly intact in the *nrpd1-50* N-terminus mutant (Supplementary Figure S6).

Sites of residual methylation included *AtSN1, Copia, MuDR* and *Helitron* elements at loci across all five Arabidopsis chromosomes. Nine such hypo-DMRs were validated by Chop-PCR (Figure 5E). For these assays, genomic DNA from WT or mutant plants was digested with a methylation-sensitive restriction enzyme, then PCR was performed spanning the enzyme's recognition sites. Successful PCR indicated that the template DNA was methylated (protected), whereas weaker amplification indicated that little or no DNA methylation was present. The nrpd1-50 mutant displayed somewhat less CHH methylation than WT SucSul, but methylation was nearly undetectable at these sites in the *nrpd1-51* null mutant (Figure 5E). We hypothesize that the Pol IV*^nrpd1-50^*enzyme continues to target many TEs in the Arabidopsis genome. However, because siRNA biogenesis is impaired, Pol IV*^nrpd1-50^-*RdDM only partially methylates these loci.

### Residual RdDM and the loss of genome surveillance in NRPD1 N-terminus mutants

**Figure 6.**
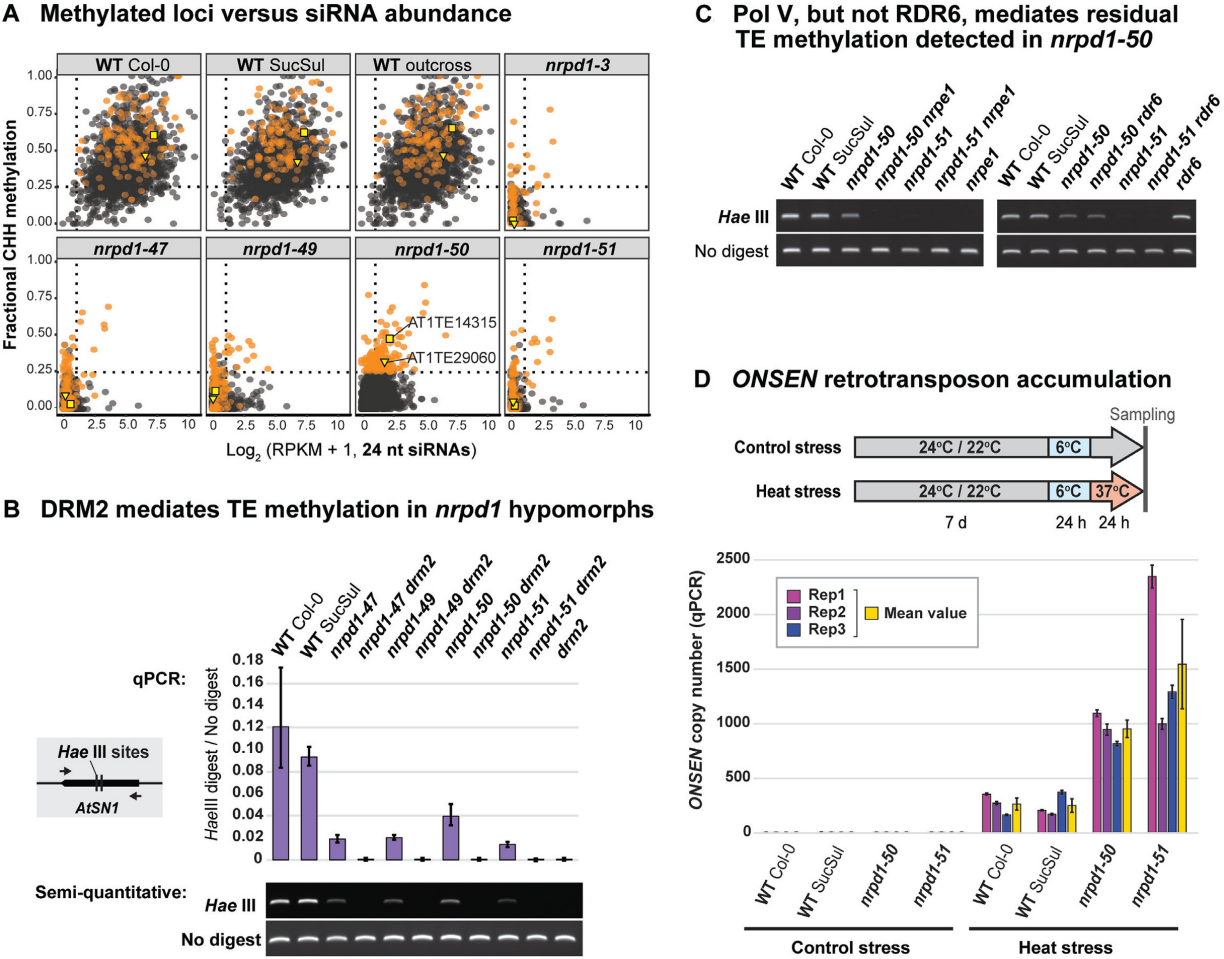
Residual Pol IV, DRM2 and Pol V-dependent genome surveillance in *nrpd1* hypomorphs. (**A**) Methylated loci versus siRNA abundance. Fractional CHH methylation (*y*-axis) is shown plotted versus 24 nt siRNA abundance (*x*-axis) at each Pol IV-RdDM locus. The dotted lines enclose 98% of all loci in the *nrpd1-51* subplot, demarcating a complete loss of Pol IV-RdDM. The orange dots represent loci that display residual methylation in the nrpd1-50 mutant. The yellow square and triangle represent AT1TE14315 and AT1TE29060, respectively, two TEs showing residual methylation validated by Chop-PCR (Figure 5E). (**B**) DRM2 mediates TE methylation in *nrpd1* hypomorphs. Chop-PCR assays at the *AtSN1* locus using the *Hae* III methylation-sensitive restriction enzyme, followed by either qPCR (above) or semi-quantitative PCR (below). Mutant alleles of *nrpd1* were tested alongside double mutant combinations with the *drm2* null mutant. ‘No digest’ controls are PCR products from assays omitting the restriction enzyme. (**C**) Pol V, but not RDR6, mediates residual TE methylation detected in *nrpd1-50*. Chop-PCR assays at the *AtSN1* locus, like in panel B, except using *nrpd1* double mutant combinations with *nrpe1* (*pol V*) or *nrpd1* double mutant combinations with *rdr6*. (**D**) *ONSEN* retrotransposon accumulation. Above: Design of the assay: ‘control stress’ plants were grown at 24°C/22°C (day/night) with a single 24 h exposure to 6°C, whereas ‘heat stress’ plants were grown at 24°C/22°C, exposed for 24 h to 6°C, then heat-stressed for 24 h at 37°C. Below: Extrachromosomal *ONSEN* accumulation measured by qPCR for WT (Col-0 and SucSul), *nrpd1-50* and *nrpd1-51* plants grown under control and heat stress conditions. Error bars indicate the standard error of the mean for the three qPCR technical replicates used to analyze each biological replicate.

We considered three alternatives to the working hypothesis that residual siRNAs and methylation in *nrpd1-50* stem from partially operative Pol IV-RdDM. First, these differentially methylated loci could be epigenetic variants segregating independently of Pol IV function. This alternative predicts that outcrossing a *pol IV* mutation to Col-0 would fail to restore siRNA production and methylation. To test this prediction, we plotted 24 nt siRNA abundance (x-axis) versus CHH methylation (*y*-axis) at all putative Pol IV targets and compared the WT Col-0, SucSul and outcross controls to *nrpd1* mutants (Figure 6A). All the WT samples showed Pol IV targets ranging from high siRNA and methylation levels (Figure 6A, top right-hand) to moderate siRNA and methylation levels (Figure 6A, dotted lines). Over 135 targets showed high (>25%) fractional CHH methylation in *nrpd1-50* plants. These loci (Figure 6A, orange points) were consistently methylated in WT Col-0, SucSul and outcross samples, and the vast majority dropped below 25% fractional methylation in nrpd1-3 and *nrpd-51* null mutants, indicating that prominent loci showing residual methylation in *nrpd1-50* are *bone fide* Pol IV targets (Figures 6A). For example, *META1* retrotransposons (e.g. AT1TE14315) occupy coordinates at the top right-hand of the WT plots, signifying high siRNA and CHH methylation levels. These TEs are depleted of siRNAs/CHH methylation in *nrpd1-3* and *nrpd1-51* null mutants but display residual siRNAs/CHH methylation in *nrpd1-50* (Figure 6A, yellow markers). These data are all consistent with Pol IV*^nrpd1-50^* mediating limited RdDM at certain TEs.

A second alternative to our working hypothesis is that Pol IV deficiency in *nrpd1* N-terminus mutants is compensated by another DNA methyltransferase: i.e. instead of the Pol IV*^nrpd1-50^*-Pol V-DRM2 machinery, we might be detecting an ectopic, *de novo* DNA methylation activity of CMT3 (61). To test which methyltransferase is required for CHH methylation in *nrpd1* N-terminus mutants, we crossed *nrpd1-47, nrpd1-49, nrpd1-50* and *nrpd1-51*, respectively, to *drm2* and *cmt3* null alleles. The CHH methylation detected in *nrpd1* N-terminus hypomorphs was lost in *nrpd1 drm2* double mutants (Figure 6B and Supplementary Figure S7A) but persisted in *nrpd1 cmt3* double mutants (Supplementary Figure S7B), showing that this residual methylation is primarily DRM2-dependent. Analysis of *nrpd1-50 nrpe1* and *nrpd1-51 nrpe1* double mutants confirmed that Pol V is also required for the residual methylation in nrpd1-50 (Figure 6C and Supplementary Figure S7C; left-hand panels).

**Figure 7.**
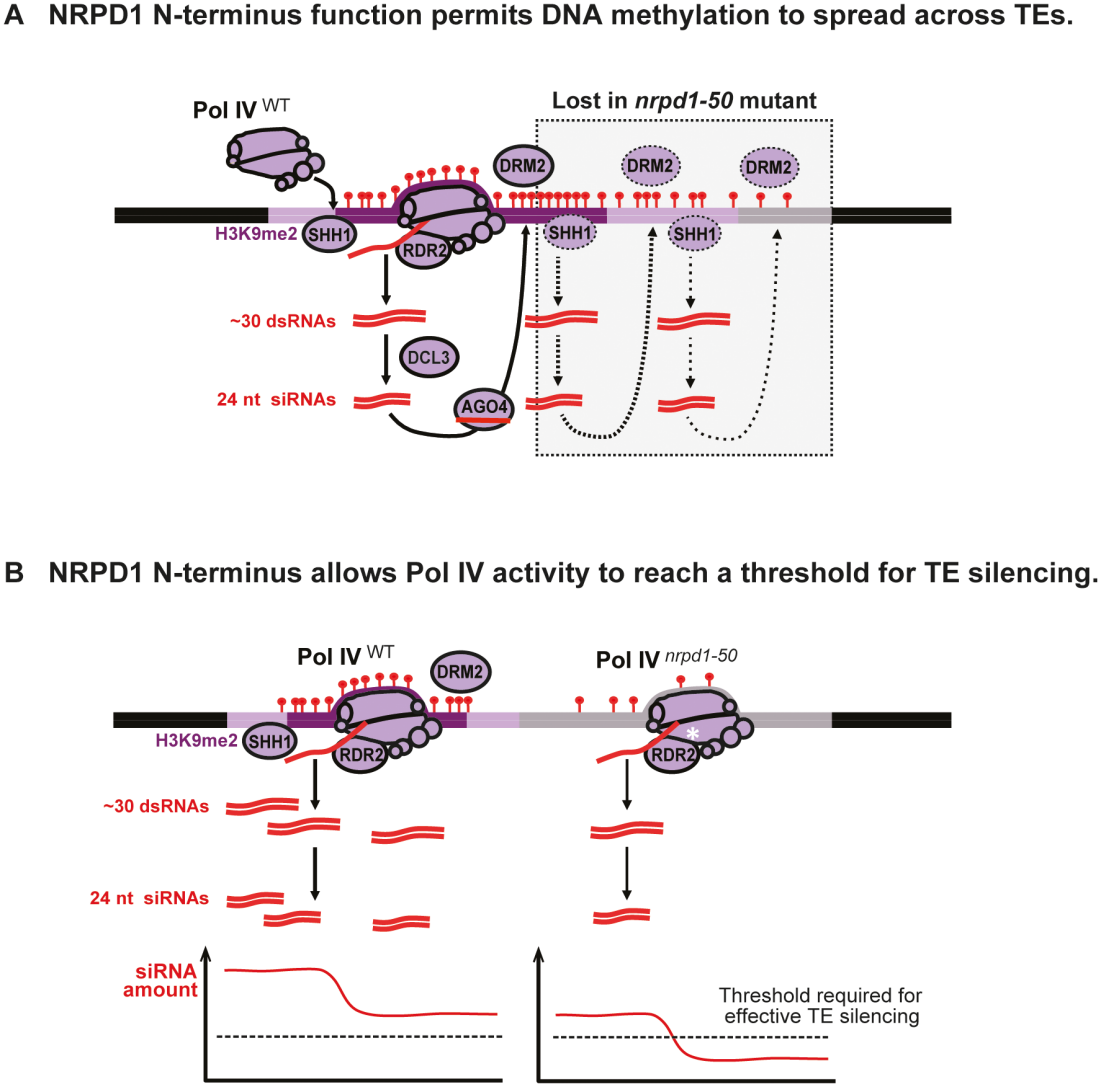
Distinct models for NRPD1 N-terminus function in genome surveillance. Pol IV can be recruited to chromosomal targets via an accessory protein, SHH1, that recognizes dimethylated histone 3 lysine 9 (H3K9me2) (31). Pol IV and its partner enzyme RDR2 synthesize ∼30 bp dsRNAs, which are cleaved by DCL3 into 24 nt siRNAs (6,8–10). AGO4 loaded with a 24 nt siRNA then guides *de novo* DNA methylation (red markers) through Pol V and DRM2 (14,15). (**A**) In one model, RdDM feeds forward, amplifies and spreads across TEs, leading to robust DNA methylation patterning in WT plants (Pol IV^WT^). In plants expressing Pol IV*^nrpd1-50^*, by contrast, RdDM initiates at particular regions but is unable spread across TEs (gray box), providing for only sporadic siRNA production. With the NRPD1 N-terminus disrupted, the residual DNA methylation in *nrpd1-50* plants is thus insufficient to maintain genome surveillance. (**B**) In an alternative model, Pol IV-dependent siRNA biogenesis requires a fully functional NRPD1 N-terminus (Pol IV^WT^) in order to reach the threshold for robust RdDM and TE silencing. In plants expressing Pol IV*^nrpd1-50^*, the overall siRNA accumulation (thin red curve) is frequently below this threshold (dotted black line), leading to dramatic but variable DNA methylation losses and reduced genome surveillance in *nrpd1-50* plants.

Having established that DRM2 and Pol V are required for residual DNA methylation in *nrpd1-50*, there remained a third alternative. Non-canonical RdDM can occur when Pol II transcription of an active TE generates substrates for the enzyme RDR6. dsRNA precursors produced in this alternative pathway are diced into 21 nt siRNAs, rather than 24 nt siRNAs, which may guide TE transcript cleavage and/or DNA methylation (37,39). Arguing against this alternative here, siRNAs in the *nrpd1-50* mutant were not predominantly 21 nt in length (Supplementary Figure S3) and the methylation persisted in *nrpd1-50 rdr6* double mutants (Figure 6C and Supplementary Figure S7C; right-hand panels). Instead, we propose that Pol IV*^nrpd1-50^* mediates canonical RdDM restricted to TE subfeatures, either because the Pol IV motif disrupted in *nrpd1-50* is required for methylation to spread across TEs, or because the threshold quantity of siRNAs needed for localized RdDM can still be produced at certain subfeatures despite the *nrpd1-50* mutation (see Figure 7, models).

DNA methylation facilitated by the NRPD1 N-terminus could be key to preventing TE proliferation. To test whether the Pol IV-specific motif is required to prevent retrotransposon activity, we measured the accumulation of extrachromosomal *ONSEN* DNA in plants exposed to heat stress (24 h at 37°C) and compared these levels to plants treated with control stress. Under control conditions WT and *nrpd1* plants showed the same low *ONSEN* copy number (Figure 6D, left-hand panel). By contrast, the *nrpd1-50* N-terminus mutant displayed a 4-fold higher *ONSEN* copy number than WT Col-0 or SucSul plants (Figure 6D, right-hand panel). *ONSEN* accumulation in *nrpd1-51* null plants was even higher than *nrpd1-50* when averaged over three biological replicates (Figure 6D, yellow bars). In conclusion, despite residual traces of Pol IV-RdDM in *nrpd1-50* plants, the Pol IV signature motif in the NRPD1 N-terminus is absolutely critical for genome surveillance.

## DISCUSSION

Pol IV transcribes chromosomal DNA into primary precursors for siRNAs that guide TE methylation in plants (6,8,9). Until now, little was known about novel domains in the Pol IV core that could govern this specialized function. The Pol IV active center undoubtedly includes NRPD1 ‘Domain D’ with its aspartate triad orthologous to the Pol II residues that coordinate Mg^2+^ for catalysis of phosphodiester bonds in RNA (48,49,57); however, like Pol V, Pol IV has deletions impacting the ‘trigger loop’ and ‘bridge-helix’ subdomains that are found in Pol II and most other multisubunit RNA polymerases (9,48) (see Supplementary Figure S4). The latter NRPD1 sequence polymorphisms explain why Pol IV is α-amanitin insensitive and likely contribute to its high error rate (9,36,62), but they are not alterations exclusive to the Pol IV enzyme. Our present study reveals that the NRPD1 N-terminus harbors a motif that is uniquely conserved in Pol IV (i.e. absent in Pols I/II/III/V) and required for robust 24 nt siRNA biogenesis, RdDM and genome surveillance.

Remarkably, plants expressing the Pol IV*^nrpd1-50^*-RDR2 complex produce 55 times fewer 24 nt siRNAs at Pol IV-RdDM targets than observed in WT plants (see Figure 3C). Despite being undetectable by northern blot, these trace siRNAs appear to be sufficient to direct CHH methylation to sites throughout the Arabidopsis genome. The presence of 24 nt siRNA peaks at TE extremities and a limited number of other hotspots in *nrpd1-50* (see Figure 5D; Supplementary Figures S5 and S6) suggests that these sites are regions of RdDM initiation from which an amplified genome surveillance response could expand. One possible model is that the Pol IV-specific motif that we discovered in the NRPD1 N-terminus governs such a process in WT plants. RNA-induced transcriptional silencing (RITS) in *Schizosaccharomyces pombe* resembles plant RdDM in many respects. During RITS, positive feedback couples siRNA biogenesis to H3K9 methylation and drives *cis*-spreading of heterochromatin across repeats (63). An analogous mechanism could potentially facilitate Pol IV function in plant genome surveillance (Figure 7A).

In WT Arabidopsis, one of several RNA-triggered initiation mechanisms (37,38,64,65) could seed H3K9me2 for SHH1-based Pol IV recruitment. The coupled activities of Pol IV and RDR2 are known to generate ∼30 bp dsRNAs that are diced into 24 nt siRNAs corresponding to the DNA template (6,8–10). After siRNAs guide AGO4 to Pol V scaffold transcripts (14), positive feedback in the SHH1-Pol IV-Pol V-DRM2 system could promote RdDM spreading across TEs (Figure 7A). Supporting this ‘spreading model’, we found that the *nrpd1-50* mutation attenuates RdDM without disrupting Pol IV-RDR2 assembly, leaving TE subfeatures still targeted by the Pol IV*^nrpd1-50^*-Pol V-DRM2 machinery *in vivo*. An alternative ‘threshold model’, however, could explain sites of methylation loss in *nrpd1-50* as sequences that are less subject to RdDM in WT plants. Loss of RdDM would occur where siRNA steady-state levels drop below a putative threshold needed to target AGO4 to Pol V transcripts for DRM2 recruitment (Figure 7B). The lower WT level of 24 nt siRNAs arising from long TE bodies compared to TE edges (26,66) could make RdDM targeting these TE bodies more sensitive to the *nrpd1-50* allele (e.g., *Copia* body versus LTRs, see Figure 5).

To balance genome surveillance with growth and development, DNA methylation must be deposited and maintained on TEs without spreading to the transcription start sites of essential genes (3,67). The selective, reliable and mutually exclusive recruitment of Pol II and Pol V to defined sequences and chromatin states helps to define otherwise fluid boundaries between TEs and genes (13,25,68,69). Modulating Pol IV activity within the confines of RdDM targets could provide an additional safeguard by delimiting regions of 24 nt siRNA biogenesis (Figure 7A). Nevertheless, the mechanisms that negatively regulate Pol IV to prevent deleterious spreading of RdDM remain unclear. A potential Pol IV regulatory function for the NRPD1 N-terminus motif (see Figure 4) should be explored. Moreover, SHH1 and CLSY proteins enhance Pol IV activity *in vivo* (31,34), and could thus permit siRNA levels to reach the putative threshold for TE silencing of the alternative model (Figure 7B). Further studies will be needed to determine whether the NRPD1 N-terminus motif is required for Pol IV partnerships with SHH1 or CLSY proteins, for Pol IV recruitment, or for other steps such as transcription start-site scanning, initiation or elongation.

## DATA AVAILABILITY

Next-generation sequencing data generated for this study have been deposited in the NCBI Sequence Read Archive (http://www.ncbi.nlm.nih.gov/sra) under accession number PRJNA510791.

## Supplementary Material

gkz618_Supplemental_Files

## Notes

Present address: Michael Thieme, Department of Plant & Microbial Biology, Universität Zürich, CH-8008 Zürich, Switzerland.

Present address: Etienne Bucher, Agroscope, Research Division Plant Breeding, CH-1260 Nyon, Switzerland.
