# Supplementary material for: The NRPD1 N-terminus contains a Pol IV-specific motif that is critical for genome surveillance in Arabidopsis
Source: Nucleic Acids Res. 2019 Aug 2;47(17):9037–52. doi: 10.1093/nar/gkz618 (PMC6753494; doi:10.1093/nar/gkz618)
Supplement: gkz618_Supplemental_Files [file gkz618_supplemental_files.zip › Ferrafiat_2019_RevisedSupplTables.pdf]

Table S01. Oligonucleotide primer and probe sequences

Primer names and sequences used in this study for genotyping, RT-qPCR, semiquantivative PCR, qPCR, bisulfite sequencing, small RNA probes and activity

1. Genotyping *nrpd1* point mutations

Genotyping assay	Oligo name	Sequence
CAPS with *Pst*I	nrpd1-47_PstICAPS_F1	AAATGTCTGTGCTAGAGGTTGAAGC
	nrpd1-47_PstICAPS_R1	GCGAAGTTTATAACCCCGAAATGC
CAPS with *Mse*I	nrpd1-48_MseICAPS_F1	CAGAACCTGTGGCAGCAAAGATCG
	nrpd1-48_MseICAPS_R1	ACAACGCAGCTACCTCCTTGAG
CAPS with *Taq*I	nrpd1-49_TaqICAPS_F1	GTTTGCGAAGGTATCATAAAGAATCTGG
	nrpd1-49_TaqICAPS_R1	GTTTAACAACGCAGCTACCTCCTTGAGGAAATACGGGTTTATTAACG
dCAPS with *Rsa*I	nrpd1-50_RsaICAPS_F1	CTGTCCGGGGTGTAAATACATTCG
	nrpd1-50_RsaICAPS_R1	AGTGTTGGAACAAAGTTTTAAACTGAGAAAATTAGTTGCAATCATATATTCTTACAGTGCAGTATGTA
dCAPS with *Xba*I	nrpd1-51_XbaICAPS_F1	GATATCATACGGGTTGCGGGAAGCTGAGCAAGTTTGCAACAAGCAACAGCTAATGGTTGAATCTAG
	nrpd1-51_XbaICAPS_R1	CTCTGTAAGCATCCTTAAAAGCACTGAC
Sanger sequencing	NRPD1_RegionA_F1	ATGGAAGACGATTGTGAGGAGC
	NRPD1_RegionA_R1	CTTCGTTTACTTCAACAACGATTCC
Sanger sequencing	nrpd1-51_seq_F2	GATCTGCTTGGCTCAGAGATG
	nrpd1-51_seq_R2	CTGTTCGGGTCATTCCAAGC

2. Reverse Transcription quantitative PCR

Target	Oligo name	Sequence
AtSN1 RT-qPCR	AtSN1_qRT_A122_F01	CCAGAAATTCATCTTCTTTGGAAAAG
	AtSN1_qRT_A123_R01	GCCCAGTGGTAAATCTCTCAGATAGA
ACTIN2 RT-qPCR	ACT2_qRT_F	CTTGCACCAAGCAGCATGAA
	ACT2_qRT_R	CCGATCCAGACACTGTACTTCCTT

3. Semiquantitative PCR

Amplicon type	Oligo name	Sequence
AtSN1 RT-PCR, Chop-PCR	AtSN1_A_F	ACCAACGTGTTGTTGGCCCAGTGGTAA
	AtSN1_A_R	AAAATAAGTGGTGGTTGTACAAGC
ACTIN2 RT-PCR	ACT2_RT_F4	GTATGATGAGGCAGGTCCAGG
	ACT2_RT_R4	ATTAACATTGCAAAGAGTTTCAAGG
Chop-PCR	AT1TE14315_C_b_F1	AGTCCACAGTGAAGCATATATGA
	AT1TE14315_C_b_R1	GGGAGTAATTGTATGTTGCACTCT
Chop-PCR	AT1TE14315_C_a_F1	ACCTTTTAGAGCGTGCTGAT
	AT1TE14315_C_a_R1	ACTCTTGCTTGTCTCATACTCTCT
Chop-PCR	AT4TE32060_C_F1	TTTTGGAGCAAATTGACTTATACGA
	AT4TE32060_C_R1	ATGAGTGTGTTCTTCCATTGAGA
Chop-PCR	AT1TE29060_C_F1	TACCTCAGGAAAACGCCGC
	AT1TE29060_C_R1	TAACGGAACCGAATCGCGTC
Chop-PCR	AT2TE24335_C_F1	ATCTTTCTCGATCACGCGCC
	AT2TE24335_C_R1	TGAAATCGGAGAGAAAATTCGCC
Chop-PCR	AT3TE90030_C_F1	TACAGCCGCACTTCTCAAAGT
	AT3TE90030_C_R1	AACAGTTGCCTAAATTTTGGTCA
Chop-PCR	AT3TE40900_C_F1	ACATGCTATTTTGAAAATTTACACGGA
	AT3TE40900_C_R1	ACGAACATTGCCAACAAAGCT
Chop-PCR	soloLTR-C_F	ATAAAACTCGAAACAAGAGTTTTCTTA
	soloLTR-C_R	TAATGGTATTATTTTGATCAGTGTTAT

4. qPCR

Amplicon type	Oligo name	Sequence
AtSN1 Chop-qPCR	AtSN1_A_F	ACCAACGTGTTGTTGGCCCAGTGGTAA
	AtSN1_A_R	AAAATAAGTGGTGGTTGTACAAGC
ONSEN qPCR	ONSEN_qPCR_F	CCACAAGAGGAACCAACGAA
	ONSEN_qPCR_R	TTCGATCATGGAAGACCGG
	ONSEN_probe	(FAM) AAGTCGGCAATAGCTTTGGCGAAGA (BHQ1)
ACTIN2 qPCR	ACT2_qPCR_F	TGCCAATCTACGAGGGTTTC
	ACT2_qPCR_R	TTACAATTTCCCGCTCTGCT
	ACT2_probe	(JOE) TCCGTCTTGACCTTGCTGGACG (BHQ1)

5. Bisulfite sequencing

PCR amplicon	Oligo name	Sequence
AtSN1 bisulfite-seq	AtSN1-Bi-F	GTTGTATAAGTTTAGTTTTAATTTTAYGGATYAGTATTAATTT
	AtSN1-Bi-R	CAATATACRATCCAAAAAACARTTATTAAAATAATATCTTAA
Bisulfite-seq controls	BScontrol1F	CGTCTGGTGATTCACCCACTTCTGTTCTCAACG
	BScontrol2F	TGTTTGGTGATTTATTTATTTTTGTTTTTAATG
	BScontrolR	CTCTCACTTTCTATCCCATTCTA

6. Small RNA blot probes

Target	Oligo name	Sequence
AtREP2 siRNAs	AtREP2 probe	GCGGGACGGGTTTGGCAGGACGTTACTTAAT
SIMPLEHAT2 siRNAs	SIMPLEHAT2 probe	TGGGTTACCCATTTTGACACCCCTA
META1 Copia LTR siRNAs	Meta1_LTR_probe	GCCCATCATCTAAGCCCATCATCT
AtSN1 siRNAs	AtSN1 probe1	CACCAACGTGTTGTTGGCCCAGTGGTAAATCTCTCAGATAGAGGTGCTGGATTCGAGACA
	AtSN1 probe2	CTCTCAGATAGAGGTGCTGGATTCGAGACACGTTGGGAAGGATCTCTTTTCCAAAGAAGA
	AtSN1 probe3	CGTTGGGAAGGATCTCTTTTCCAAAGAAGATGAATTTCTGGTATGGGTCCCGCCTCTGGG
	AtSN1 probe4	ATTTCTGGTATGGGTCCCGCCTCTGGGAGATGTAAGGCCTTTGGGCCTGAACTTCCAG

7. Activity assays

	Oligo name	Sequence
Tripartite substrate	DNA Template	CTAATTCGAGTCAGTCAACGAAAGCTGACTGTGTACGCCTGGTCCGACTCG
	RNA Primer	GCAGCTTTCGTTGACTGACTCGAATTAG
	RNA Primer	rUrGrCrArUrArArArGrArCrCrArGrGrC

Table S02. MethylC-seq read data

Table showing filenames and total number of sequence from bisulfite sequencing for: WT Col-0, WT SucSul and WT outcross controls; the point mutants *nrpd1-47*, *nrpd1-49*, *nrpd1-50* and *nrpd1-51*; and the T-DNA mutants *nrpd1-3* and *nrpe1-11*. Data were collected as two replicates per genotype, with read1 (R1) and read2 (R2) of the 2x150 Illumina HiSeq run provided in separate FastQ files.

Sample	Filename	Encoding	Total Sequences	Sequence length
mcseq_01_WT_Col0_rep1_R1	mcseq_01_WT_Col0_rep1_R1.fq.gz	Sanger / Illumina 1.9	18 802 219	150
mcseq_01_WT_Col0_rep1_R2	mcseq_01_WT_Col0_rep1_R2.fq.gz	Sanger / Illumina 1.9	18 802 219	150
mcseq_02_WT_SucSul_rep1_R1	mcseq_02_WT_SucSul_rep1_R1.fq.gz	Sanger / Illumina 1.9	19 157 496	150
mcseq_02_WT_SucSul_rep1_R2	mcseq_02_WT_SucSul_rep1_R2.fq.gz	Sanger / Illumina 1.9	19 157 496	150
mcseq_03_nrpd1_47_rep1_R1	mcseq_03_nrpd1_47_rep1_R1.fq.gz	Sanger / Illumina 1.9	18 846 495	150
mcseq_03_nrpd1_47_rep1_R2	mcseq_03_nrpd1_47_rep1_R2.fq.gz	Sanger / Illumina 1.9	18 846 495	150
mcseq_04_nrpd1_49_rep1_R1	mcseq_04_nrpd1_49_rep1_R1.fq.gz	Sanger / Illumina 1.9	19 097 120	150
mcseq_04_nrpd1_49_rep1_R2	mcseq_04_nrpd1_49_rep1_R2.fq.gz	Sanger / Illumina 1.9	19 097 120	150
mcseq_05_nrpd1_50_rep1_R1	mcseq_05_nrpd1_50_rep1_R1.fq.gz	Sanger / Illumina 1.9	19 001 928	150
mcseq_05_nrpd1_50_rep1_R2	mcseq_05_nrpd1_50_rep1_R2.fq.gz	Sanger / Illumina 1.9	19 001 928	150
mcseq_06_nrpd1_51_rep1_R1	mcseq_06_nrpd1_51_rep1_R1.fq.gz	Sanger / Illumina 1.9	18 818 859	150
mcseq_06_nrpd1_51_rep1_R2	mcseq_06_nrpd1_51_rep1_R2.fq.gz	Sanger / Illumina 1.9	18 818 859	150
mcseq_07_WT_outx_rep1_R1	mcseq_07_WT_outx_rep1_R1.fq.gz	Sanger / Illumina 1.9	18 789 905	150
mcseq_07_WT_outx_rep1_R2	mcseq_07_WT_outx_rep1_R2.fq.gz	Sanger / Illumina 1.9	18 789 905	150
mcseq_08_nrpd1_3_rep1_R1	mcseq_08_nrpd1_3_rep1_R1.fq.gz	Sanger / Illumina 1.9	18 665 931	150
mcseq_08_nrpd1_3_rep1_R2	mcseq_08_nrpd1_3_rep1_R2.fq.gz	Sanger / Illumina 1.9	18 665 931	150
mcseq_09_nrpe1_11_rep1_R1	mcseq_09_nrpe1_11_rep1_R1.fq.gz	Sanger / Illumina 1.9	18 768 862	150
mcseq_09_nrpe1_11_rep1_R2	mcseq_09_nrpe1_11_rep1_R2.fq.gz	Sanger / Illumina 1.9	18 768 862	150
mcseq_10_WT_Col0_rep2_R1	mcseq_10_WT_Col0_rep2_R1.fq.gz	Sanger / Illumina 1.9	19 181 629	150
mcseq_10_WT_Col0_rep2_R2	mcseq_10_WT_Col0_rep2_R2.fq.gz	Sanger / Illumina 1.9	19 181 629	150
mcseq_11_WT_SucSul_rep2_R1	mcseq_11_WT_SucSul_rep2_R1.fq.gz	Sanger / Illumina 1.9	18 638 666	150
mcseq_11_WT_SucSul_rep2_R2	mcseq_11_WT_SucSul_rep2_R2.fq.gz	Sanger / Illumina 1.9	18 638 666	150
mcseq_12_nrpd1_47_rep2_R1	mcseq_12_nrpd1_47_rep2_R1.fq.gz	Sanger / Illumina 1.9	18 943 428	150
mcseq_12_nrpd1_47_rep2_R2	mcseq_12_nrpd1_47_rep2_R2.fq.gz	Sanger / Illumina 1.9	18 943 428	150
mcseq_13_nrpd1_49_rep2_R1	mcseq_13_nrpd1_49_rep2_R1.fq.gz	Sanger / Illumina 1.9	18 691 510	150
mcseq_13_nrpd1_49_rep2_R2	mcseq_13_nrpd1_49_rep2_R2.fq.gz	Sanger / Illumina 1.9	18 691 510	150
mcseq_14_nrpd1_50_rep2_R1	mcseq_14_nrpd1_50_rep2_R1.fq.gz	Sanger / Illumina 1.9	18 570 988	150
mcseq_14_nrpd1_50_rep2_R2	mcseq_14_nrpd1_50_rep2_R2.fq.gz	Sanger / Illumina 1.9	18 570 988	150
mcseq_15_nrpd1_51_rep2_R1	mcseq_15_nrpd1_51_rep2_R1.fq.gz	Sanger / Illumina 1.9	18 833 670	150
mcseq_15_nrpd1_51_rep2_R2	mcseq_15_nrpd1_51_rep2_R2.fq.gz	Sanger / Illumina 1.9	18 833 670	150
mcseq_16_WT_outx_rep2_R1	mcseq_16_WT_outx_rep2_R1.fq.gz	Sanger / Illumina 1.9	18 935 317	150
mcseq_16_WT_outx_rep2_R2	mcseq_16_WT_outx_rep2_R2.fq.gz	Sanger / Illumina 1.9	18 935 317	150
mcseq_17_nrpd1_3_rep2_R1	mcseq_17_nrpd1_3_rep2_R1.fq.gz	Sanger / Illumina 1.9	18 591 301	150
mcseq_17_nrpd1_3_rep2_R2	mcseq_17_nrpd1_3_rep2_R2.fq.gz	Sanger / Illumina 1.9	18 591 301	150
mcseq_18_nrpe1_11_rep2_R1	mcseq_18_nrpe1_11_rep2_R1.fq.gz	Sanger / Illumina 1.9	18 745 824	150
mcseq_18_nrpe1_11_rep2_R2	mcseq_18_nrpe1_11_rep2_R2.fq.gz	Sanger / Illumina 1.9	18 745 824	150

Table S03. Bismark analysis from mapped MethylC-seq data

The table some statistics on the mapping process to the TAIR10 reference genome and the percentages of methylated cytosines (in CG, CHG and CHH contexts) for each sample and the two replicates: WT Col-0, WT SucSul, and WT outcross controls; the point mutants *nrpd1-47*, *nrpd1-49*, *nrpd1-50,* and *nrpd1-51*; and the T-DNA mutants *nrpd1-3* and *nrpe1-11*.

Sample	Total unmethylated C's in CG context	Total unmethylated C's in CHH context	Total unmethylated C's in CHG context	Total number of C's analysed	Total methylated C's in CG context	Total methylated C's in CHH context	Total methylated C's in CHG context	Sequence pairs with no alignments under any condition
**mcseq_01_WT_Col0_rep1_R1_val_1_bismark_PE_report**	67809738	451291448	88588595	650900197	22757838	10762628	9689950	4818698
**mcseq_02_WT_SucSul_rep1_R1_val_1_bismark_PE_report**	68855322	469636097	88671852	665315885	20232108	9435355	8485151	4846741
**mcseq_03_nrpd1_47_rep1_R1_val_1_bismark_PE_report**	68514241	444669444	90141141	645965951	23723200	9146771	9771154	4818468
**mcseq_04_nrpd1_49_rep1_R1_val_1_bismark_PE_report**	70089644	463477396	92141162	668381777	23931244	8983675	9758656	4695001
**mcseq_05_nrpd1_50_rep1_R1_val_1_bismark_PE_report**	68947173	454111357	90980137	654751881	22784561	8791658	9136995	4880418
**mcseq_06_nrpd1_51_rep1_R1_val_1_bismark_PE_report**	64769790	445889503	84662086	634211800	21535735	8672837	8681849	4926103
**mcseq_07_WT_outx_rep1_R1_val_1_bismark_PE_report**	67312702	429641397	88600564	631379542	24122573	10907428	10794878	5024935
**mcseq_08_nrpd1_3_rep1_R1_val_1_bismark_PE_report**	67136099	431439571	87580402	626786659	22258366	9111932	9260289	4994052
**mcseq_09_nrpe1_11_rep1_R1_val_1_bismark_PE_report**	68938878	436805541	89870833	639826066	24256905	9401150	10552759	4864742
**mcseq_10_WT_Col0_rep2_R1_val_1_bismark_PE_report**	69928597	474737321	90947504	678054736	22370763	10713946	9356605	4591665
**mcseq_11_WT_SucSul_rep2_R1_val_1_bismark_PE_report**	65992216	438749571	86322109	633711792	22301382	10633151	9713363	4865304
**mcseq_12_nrpd1_47_rep2_R1_val_1_bismark_PE_report**	68019925	457803258	88987008	653666243	21569166	8699776	8587110	4817209
**mcseq_13_nrpd1_49_rep2_R1_val_1_bismark_PE_report**	63967170	424072792	84497703	611418427	21122299	9098066	8660397	5563854
**mcseq_14_nrpd1_50_rep2_R1_val_1_bismark_PE_report**	65603259	439462016	86671121	630955411	21314016	9344050	8560949	5022175
**mcseq_15_nrpd1_51_rep2_R1_val_1_bismark_PE_report**	65984115	439562058	86415383	631155396	21702925	8748414	8742501	5127995
**mcseq_16_WT_outx_rep2_R1_val_1_bismark_PE_report**	65788518	450462957	86032486	644803249	22025560	10780379	9713349	4849620
**mcseq_17_nrpd1_3_rep2_R1_val_1_bismark_PE_report**	65695355	415553239	85930299	609651644	22977427	9673509	9821815	5234658
**mcseq_18_nrpe1_11_rep2_R1_val_1_bismark_PE_report**	68460294	442271316	89365210	641397800	22625876	9031697	9643407	4832506

Bismark command and options:

I. Bismark mapping	bismark --rg_tag --rg_id $sample_id --rg_sample $sample_name --sam --bowtie1 --nucleotide_coverage --samtools_path /biotools/samtools/1.5/ --unmapped --non_bs_mm --ambigu[illegible]
II. Bismark deduplication	deduplicate_bismark --paired ${sam}
III. Bismark methylation extractor	bismark_methylation_extractor --paired-end --ignore_r2 5 --no_header --output $output_dir --bedGraph --CX_context --buffer_size 10G --cytosine_report --CX_context --genome_fold[illegible]

Sequence pairs which were discarded because genomic sequence could not be extracted	Sequence pairs did not map uniquely	Sequence pairs analysed in total	Number of paired-end alignments with a unique best hit	Number of alignments to (merely theoretical) complementary strands being rejected in total	Mapping efficiency	C methylated in CG context	C methylated in CHH context	C methylated in CHG context	Sample2
5	1163881	18564116	12581537	0	67.80%	25.10%	2.30%	9.90%	**mcseq_01_WT_Col0_rep1_R1_val_1_bismark_PE_report**
3	1088280	18857076	12922055	0	68.50%	22.70%	2.00%	8.70%	**mcseq_02_WT_SucSul_rep1_R1_val_1_bismark_PE_report**
5	1238792	18509796	12452536	0	67.30%	25.70%	2.00%	9.80%	**mcseq_03_nrpd1_47_rep1_R1_val_1_bismark_PE_report**
2	1191153	18806837	12920683	0	68.70%	25.50%	1.90%	9.60%	**mcseq_04_nrpd1_49_rep1_R1_val_1_bismark_PE_report**
3	1123408	18717547	12713721	0	67.90%	24.80%	1.90%	9.10%	**mcseq_05_nrpd1_50_rep1_R1_val_1_bismark_PE_report**
9	1082150	18514442	12506189	0	67.50%	25.00%	1.90%	9.30%	**mcseq_06_nrpd1_51_rep1_R1_val_1_bismark_PE_report**
5	1197660	18472890	12250295	0	66.30%	26.40%	2.50%	10.90%	**mcseq_07_WT_outx_rep1_R1_val_1_bismark_PE_report**
6	1205477	18339865	12140336	0	66.20%	24.90%	2.10%	9.60%	**mcseq_08_nrpd1_3_rep1_R1_val_1_bismark_PE_report**
4	1231926	18446207	12349539	0	66.90%	26.00%	2.10%	10.50%	**mcseq_09_nrpe1_11_rep1_R1_val_1_bismark_PE_report**
8	1132251	18953166	13229250	0	69.80%	24.20%	2.20%	9.30%	**mcseq_10_WT_Col0_rep2_R1_val_1_bismark_PE_report**
0	1125952	18347094	12355838	0	67.30%	25.30%	2.40%	10.10%	**mcseq_11_WT_SucSul_rep2_R1_val_1_bismark_PE_report**
7	1133781	18645629	12694639	0	68.10%	24.10%	1.90%	8.80%	**mcseq_12_nrpd1_47_rep2_R1_val_1_bismark_PE_report**
8	1063419	18450760	11823487	0	64.10%	24.80%	2.10%	9.30%	**mcseq_13_nrpd1_49_rep2_R1_val_1_bismark_PE_report**
3	984814	18264414	12257425	0	67.10%	24.50%	2.10%	9.00%	**mcseq_14_nrpd1_50_rep2_R1_val_1_bismark_PE_report**
4	1136344	18543002	12278663	0	66.20%	24.80%	2.00%	9.20%	**mcseq_15_nrpd1_51_rep2_R1_val_1_bismark_PE_report**
2	1053657	18680442	12777165	0	68.40%	25.10%	2.30%	10.10%	**mcseq_16_WT_outx_rep2_R1_val_1_bismark_PE_report**
4	1227429	18248176	11786089	0	64.60%	25.90%	2.30%	10.30%	**mcseq_17_nrpd1_3_rep2_R1_val_1_bismark_PE_report**
8	1189378	18466519	12444635	0	67.40%	24.80%	2.00%	9.70%	**mcseq_18_nrpe1_11_rep2_R1_val_1_bismark_PE_report**

ous --genome $genome_dir -1 $R1 -2 $R2 --fastq --phred33-quals -n1 -l 50

er $genome_dir ${sam_dedup}

Table S04. Bisulfite conversion rates for MethylC-seq data
Table showing conversion rate from chloroplastic genome alignment for the two replicates of each sample: WT Col-0, WT SucSul, and WT outcross controls; the point mutants *nrpd1-47*, *nrpd1-49*, *nrpd1-50* and *nrpd1-51*; and the T-DNA mutants *nrpd1-3* and *nrpe1-11.*

**Conversion rate**	
**Sample**	**ChrC**
**WT_Col0_rep1**	99.14%
**WT_Col0_rep2**	99.25%
**WT_SucSul_rep1**	99.22%
**WT_SucSul_rep2**	99.18%
**nrpd1-3_rep1**	99.15%
**nrpd1-3_rep2**	99.11%
**nrpd1-47_rep1**	99.17%
**nrpd1-47_rep2**	99.20%
**nrpd1-49_rep1**	99.25%
**nrpd1-49_rep2**	99.11%
**nrpd1-50_rep1**	99.23%
**nrpd1-50_rep2**	99.06%
**nrpd1-51_rep1**	99.23%
**nrpd1-51_rep2**	99.19%
**WT_outx_rep1**	99.17%
**WT_outx_rep2**	99.25%
**nrpe1-11_rep1**	99.16%
**nrpe1-11_rep2**	99.17%

Conversion_rate = (1 - (sum(methylated C)/sum(methylated + unmethylated))) * 100

Table S06. Total DMRs identified using WT SucSul as control

Table showing total counts of hypo and hyper Differentially Methylated Regions (DMRs) covering at least 100 bp for each of the following samples compared to the WT SucSul control: WT Col-0 and WT outcross; the point mutants *nrpd1-47*, *nrpd1-49*, *nrpd1-50* and *nrpd1-51*; and the T-DNA mutants *nrpd1-3* and *nrpe1-11.*

Sample	DMR-type	Context	Count
		CG	23
WT_Col0	hypo	CHG	52
		CHH	25
		CG	395
nrpd1-3	hypo	CHG	3633
		CHH	1811
		CG	26
nrpd1-47	hypo	CHG	2978
		CHH	1699
		CG	15
nrpd1-49	hypo	CHG	2548
		CHH	1626
		CG	18
nrpd1-50	hypo	CHG	1942
		CHH	1286
		CG	29
nrpd1-51	hypo	CHG	3121
		CHH	1707
		CG	14
WT_outx	hypo	CHG	26
		CHH	17
		CG	158
nrpe1-11	hypo	CHG	3089
		CHH	1843

Sample	DMR-type	Context	Count
		CG	34
WT_Col0	hyper	CHG	50
		CHH	45
		CG	56
nrpd1-3	hyper	CHG	71
		CHH	8
		CG	13
nrpd1-47	hyper	CHG	24
		CHH	8
		CG	7
nrpd1-49	hyper	CHG	24
		CHH	4
		CG	5
nrpd1-50	hyper	CHG	17
		CHH	5
		CG	6
nrpd1-51	hyper	CHG	29
		CHH	4
		CG	9
WT_outx	hyper	CHG	22
		CHH	76
		CG	63
nrpe1-11	hyper	CHG	180
		CHH	6

Table S07. Small RNA-seq read data

Table showing filenames corresponding to each sample replicate and the total number of reads from small RNA-seq libraries: WT Col-0, WT SucSul, and WT outcross controls; the point mutants *nrpd1-47*, *nrpd1-49*, *nrpd1-50,* and *nrpd1-51*; and the T-DNA mutants *nrpd1-3* and *nrpe1-11*.

Sample	Filename	Encoding	Total number of reads	Reads written (passing filters)	Reads that were too short
WT_Col0_rep1	HZQ-11_R1_015to125bp.fastq.gz	Sanger / Illumina 1.9	49520491	48894977 (98.7%)	625514 (1.3%)
WT_SucSul_rep1	HZQ-12_R1_015to125bp.fastq.gz	Sanger / Illumina 1.9	49464429	48839018 (98.7%)	625411 (1.3%)
nrpd1_47_rep1	HZQ-13_R1_015to125bp.fastq.gz	Sanger / Illumina 1.9	42323292	41740033 (98.6%)	583259 (1.4%)
nrpd1_49_rep1	HZQ-14_R1_015to125bp.fastq.gz	Sanger / Illumina 1.9	43355699	42832149 (98.8%)	523550 (1.2%)
nrpd1_50_rep1	HZQ-15_R1_015to125bp.fastq.gz	Sanger / Illumina 1.9	37426796	36901032 (98.6%)	525764 (1.4%)
nrpd1_51_rep1	HZQ-16_R1_015to125bp.fastq.gz	Sanger / Illumina 1.9	36028858	35488394 (98.5%)	540464 (1.5%)
WT_outx_rep1	HZQ-17_R1_015to125bp.fastq.gz	Sanger / Illumina 1.9	45493852	44582407 (98.0%)	911445 (2.0%)
nrpd1_3_rep1	HZQ-18_R1_015to125bp.fastq.gz	Sanger / Illumina 1.9	35107682	34408525 (98.0%)	699157 (2.0%)
nrpe1_11_rep1	HZQ-19_R1_015to125bp.fastq.gz	Sanger / Illumina 1.9	33597582	32893475 (97.9%)	704107 (2.1%)
WT_Col0_rep2	HZQ-20_R1_015to125bp.fastq.gz	Sanger / Illumina 1.9	32381564	31711441 (97.9%)	670123 (2.1%)
WT_SucSul_rep2	HZQ-21_R1_015to125bp.fastq.gz	Sanger / Illumina 1.9	47047030	46213290 (98.2%)	833740 (1.8%)
nrpd1_47_rep2	HZQ-22_R1_015to125bp.fastq.gz	Sanger / Illumina 1.9	43273939	42510667 (98.2%)	763272 (1.8%)
nrpd1_49_rep2	HZQ-23_R1_015to125bp.fastq.gz	Sanger / Illumina 1.9	30546376	29972642 (98.1%)	573734 (1.9%)
nrpd1_50_rep2	HZQ-24_R1_015to125bp.fastq.gz	Sanger / Illumina 1.9	32815056	32185130 (98.1%)	629926 (1.9%)
nrpd1_51_rep2	HZQ-25_R1_015to125bp.fastq.gz	Sanger / Illumina 1.9	39895106	39115861 (98.0%)	779245 (2.0%)
WT_outx_rep2	HZQ-26_R1_015to125bp.fastq.gz	Sanger / Illumina 1.9	44366256	43506256 (98.1%)	860000 (1.9%)
nrpd1_3_rep2	HZQ-27_R1_015to125bp.fastq.gz	Sanger / Illumina 1.9	44207968	43349959 (98.1%)	858009 (1.9%)
nrpe1_11_rep2	HZQ-28_R1_015to125bp.fastq.gz	Sanger / Illumina 1.9	47451260	46493032 (98.0%)	958228 (2.0%)

Cutadapt option "--trim-n --discard-trimmed --minimum-length 15 -q 30"

Table S08. Small RNA-seq read mapping

Small RNA-seq reads were mapped to the TAIR10 reference using bowtie v1.2.2. The table shows a summary of mapping results for the two replicates of each sample: WT Col-0, WT SucSul, and WT outcross controls; the point mutants *nrpd1-47*, *nrpd1-49*, *nrpd1-50,* and *nrpd1-51*; and the T-DNA mutants *nrpd1-3* and *nrpe1-11*.

Sample	Filename	Reads processed	Reads aligned	Reads aligned percentage	Reads not aligned	Reads not aligned percentage
WT_Col0_rep1	HZQ-11_R1_015to125bp.fastq.gz	48894977	41210919	84.28	7684058	15.72
WT_SucSul_rep1	HZQ-12_R1_015to125bp.fastq.gz	48839018	40143601	82.2	8695417	17.8
nrpd1_47_rep1	HZQ-13_R1_015to125bp.fastq.gz	41740033	35480065	85	6259968	15
nrpd1_49_rep1	HZQ-14_R1_015to125bp.fastq.gz	42832149	34943178	81.58	7888971	18.42
nrpd1_50_rep1	HZQ-15_R1_015to125bp.fastq.gz	36901032	30885031	83.7	6016001	16.3
nrpd1_51_rep1	HZQ-16_R1_015to125bp.fastq.gz	35488394	29738073	83.8	5750321	16.2
WT_outx_rep1	HZQ-17_R1_015to125bp.fastq.gz	44582407	36269125	81.35	8313282	18.65
nrpd1_3_rep1	HZQ-18_R1_015to125bp.fastq.gz	34408525	28168918	81.87	6239607	18.13
nrpe1_11_rep1	HZQ-19_R1_015to125bp.fastq.gz	32893475	26716539	81.22	6176936	18.78
WT_Col0_rep2	HZQ-20_R1_015to125bp.fastq.gz	31711441	25935876	81.79	5775565	18.21
WT_SucSul_rep2	HZQ-21_R1_015to125bp.fastq.gz	46213290	38874142	84.12	7339148	15.88
nrpd1_47_rep2	HZQ-22_R1_015to125bp.fastq.gz	42510667	36142707	85.02	6367960	14.98
nrpd1_49_rep2	HZQ-23_R1_015to125bp.fastq.gz	29972642	25900800	86.41	4071842	13.59
nrpd1_50_rep2	HZQ-24_R1_015to125bp.fastq.gz	32185130	27475487	85.37	4709643	14.63
nrpd1_51_rep2	HZQ-25_R1_015to125bp.fastq.gz	39115861	33172931	84.81	5942930	15.19
WT_outx_rep2	HZQ-26_R1_015to125bp.fastq.gz	43506256	36177589	83.15	7328667	16.85
nrpd1_3_rep2	HZQ-27_R1_015to125bp.fastq.gz	43349959	35362924	81.58	7987035	18.42
nrpe1_11_rep2	HZQ-28_R1_015to125bp.fastq.gz	46493032	38522214	82.86	7970818	17.14

Bowtie1 parameters -v 0 -k 50 --best --strata

Table S10. Largest subunits of Pol II, Pol IV and Pol V used for multiple alignments
This table lists the species, sequence ID and source of RNA polymerase subunit sequences used for multiple protein alignments in this study.

Protein symbol	Species	Sequence ID	Source	RNA polymerase
Sc.RPB1 / RPO21	*Saccharomyces cerevisiae*	P04050	https://www.uniprot.org/	Pol II
Ath.NRPB1		P18616	https://www.uniprot.org/	Pol II
Ath.NRPD1	*Arabidopsis thaliana*	Q9LQ02	https://www.uniprot.org/	Pol IV
Ath.NRPE1		Q5D869	https://www.uniprot.org/	Pol V
Cru.NRPD1	*Capsella rubella*	Carubv10019657m	https://phytozome.jgi.doe.gov/pz/portal.html	Pol IV
Esa.NRPD1	*Eutrema salsugineum*	Thhalv10023214m	https://phytozome.jgi.doe.gov/pz/portal.html	Pol IV
Tca.NRPD1	*Theobroma cacao*	Thecc1EG016168t1	https://phytozome.jgi.doe.gov/pz/portal.html	Pol IV
Csi.NRPD1	*Citrus x sinensis*	orange1.1g000525m	https://phytozome.jgi.doe.gov/pz/portal.html	Pol IV
Lus.NRPD1	*Linum usitatissimum*	Lus10002955	https://phytozome.jgi.doe.gov/pz/portal.html	Pol IV
Csa.NRPD1	*Cucumis sativus*	Cucsa.041530.1	https://phytozome.jgi.doe.gov/pz/portal.html	Pol IV
Pvu.NRPD1	*Phaseolus vulgaris*	Phvul.002G153700	https://phytozome.jgi.doe.gov/pz/portal.html	Pol IV
Mtr.NRPD1	*Medicago truncatula*	Medtr5g011000.1	https://phytozome.jgi.doe.gov/pz/portal.html	Pol IV
Egr.NRPD1	*Eucalyptus grandis*	Eucgr.D01929.1.p	https://phytozome.jgi.doe.gov/pz/portal.html	Pol IV
Sly.NRPD1	*Solanum lycopersicum*	XP_004245657	https://www.ncbi.nlm.nih.gov/protein/	Pol IV
Sit.NRPD1	*Setaria italica*	XP_022682775	https://www.ncbi.nlm.nih.gov/protein/	Pol IV
Zma.NRPD1	*Zea mays*	GRMZM2G007681_P01	https://phytozome.jgi.doe.gov/pz/portal.html	Pol IV
Bdi.NRPD1	*Brachypodium distachyon*	XP_003566523	https://www.ncbi.nlm.nih.gov/protein/	Pol IV
Atr.NRPD1	*Amborella trichopoda*	evm_27.model.AmTr_v1.0_scaffold00069.11	https://phytozome.jgi.doe.gov/pz/portal.html	Pol IV
Gbi.NRPD1	*Ginkgo biloba*	AJA90773	https://www.ncbi.nlm.nih.gov/protein/	Pol IV
Pca.NRPD1	*Pinus canariensis*	AJA90781	https://www.ncbi.nlm.nih.gov/protein/	Pol IV

Table S11. Detection of Pol IV-specific motif in NRPD1 from other plant species

This table shows proteins found to contain the Fig. 4 motif using https://www.ebi.ac.uk/Tools/hmmer/search/hmmsearch. The DeCL domain is present and the WG repeats are missing in most cases, although C-terminal data for 7 proteins is truncated (gray-shaded).
Two NRPD1 sequences obtained as truncated records via hmmsearch (e.g., *Citrus sinensis* and *Zea mays*) were previously identified as full-length Phytozome sequences with a C-terminal DeCL domain and missing WG repeats, as expected (green-shaded).

Species	SeqCount	Putative full-length protein	DeCL (DUF3223)	WG repeat domain	Additional full-length sequences	Truncated / problematic sequences	DeCL data truncated	Domains A to H intact
Amborella trichopoda	1	none	No	No		U5D188_AMBTC	Yes	No (Domain H truncated)
Arabidopsis lyrata subsp. lyrata	1	D7KU36_ARALL	Yes	No				Yes
Arabidopsis thaliana	3	NRPD1_ARATH	Yes	No	A0A1P8ANM9_ARATH	A0A1P8ANM7_ARATH		Yes
Arabis alpina	1	A0A087HEJ6_ARAAL	Yes	No				Yes
Brachypodium distachyon	3	A0A0Q3GA61_BRADI	Yes	No	A0A2K2DBU6_BRADI	A0A2K2DBS8_BRADI		Yes
Brassica napus	1	A0A078H322_BRANA	Yes	No				Yes
Brassica oleracea var. oleracea	1	A0A0D3BVX2_BRAOL	Yes	No				Yes
Capsella rubella	1	R0HUP7_9BRAS	Yes	No				Yes, except Domain A
Cephalotus follicularis	1	A0A1Q3CGP2_CEPFO	Yes	No				Yes
Citrus clementina	1	V4TV64_9ROSI	Yes	No				Yes
Citrus x sinensis	1	see Table S10	Yes	No	orange1.1g000525m	A0A067FBP0_CITSI		Yes
Citrus unshiu	1	A0A2H5Q901_CITUN	Yes	No				Yes
Corchorus olitorius	1	A0A1R3KRE2_9ROSI	Yes	No				Yes
Cucumis melo	1	A0A1S4DY39_CUCME	Yes	No				Yes
Cucumis sativus	1	A0A0A0L2L4_CUCSA	Yes	No				Yes
Eucalyptus grandis	1	A0A059CGN4_EUCGR	Yes	No				Yes
Eutrema salsugineum	1	V4MCZ2_EUTSA	Yes	No				Yes
Gossypium barbadense	2	A0A2P5RVQ8_GOSBA	Yes	No	A0A2P5Q2J2_GOSBA			Yes, except Domain A
Gossypium hirsutum	3	A0A1U8J7Q9_GOSHI	Yes	No	A0A1U8LFL4_GOSHI A0A1U8LUJ3_GOSHI			Yes
Gossypium raimondii	2	A0A0D2N1D6_GOSRA	Yes	No	A0A0D2R845_GOSRA			Yes
Handroanthus impetiginosus	1	none	No	No		A0A2G9GK75_9LAMI	Yes	No (H domain truncated)
Hordeum vulgare subsp. vulgare	1	none	No	No		A0A287DTN4_HORVV	Yes	No (small fragment)
Juglans regia	2	A0A2I4EM69_JUGRE	Yes	No	A0A2I4EM78_JUGRE			Yes
Leersia perrieri	4	A0A0D9UXT1_9ORYZ	Yes	No	A0A0D9UXT2_9ORYZ A0A0D9UXT3_9ORYZ	A0A0D9W8E5_9ORYZ		Yes
Lupinus angustifolius	1	A0A1J7IAG7_LUPAN	Yes	No				Yes
Manihot esculenta	1	A0A251LE42_MANES	Yes	No				Yes
Medicago truncatula	1	G7KER5_MEDTR	Yes	No				Yes
Nelumbo nucifera	7	A0A1U8BAZ2_NELNU	Yes	No	A0A1U8QAR8_NELNU A0A1U8QBF8_NELNU A0A1U8QCB2_NELNU	A0A1U8BB58_NELNU A0A1U8QAS4_NELNU A0A1U8QAT1_NELNU		Yes
Oryza barthii	3	A0A0D3H9V7_9ORYZ	Yes	No	A0A0D3H9V8_9ORYZ			Yes
Oryza brachyantha	2	J3M0K5_ORYBR	Yes	No	J3MZZ4_ORYBR			Yes
Oryza glumipatula	5	A0A0E0B6C1_9ORYZ	Yes	No	A0A0E0B6C2_9ORYZ A0A0E0B6C3_9ORYZ A0A0E0B6C6_9ORYZ A0A0D9ZPI1_9ORYZ			Yes
Oryza meridionalis	3	A0A0E0DHF4_9ORYZ	Yes	No	A0A0E0DHF6_9ORYZ A0A0E0EUR1_9ORYZ			Yes
Oryza nivara	3	A0A0E0H4M5_ORYNI	Yes	No	A0A0E0ING2_ORYNI A0A0E0ING3_ORYNI			Yes, except Domain B
Oryza punctata	3	A0A0E0M4C2_ORYPU	Yes	No	A0A0E0M4C3_ORYPU A0A0E0M4C4_ORYPU			Yes
Oryza rufipogon	3	A0A0E0PCW8_ORYRU	Yes	No	A0A0E0QUX0_ORYRU A0A0E0QUX1_ORYRU			Yes, except Domain B
Oryza sativa Indica Group	2	B8BED0_ORYSI	Yes	No		B8AT49_ORYSI		Yes
Panicum hallii var. hallii	1	A0A2T7CV85_9POAL	Yes	No				Yes
Parasponia andersonii	1	A0A2P5C8X2_PARAD	Yes	No				Yes
Phaseolus vulgaris	2	V7CJT1_PHAVU	Yes	No	V7CM57_PHAVU			Yes
Phoenix dactylifera	2	A0A2H3ZWV6_PHODC	Yes	No	A0A2H3ZW74_PHODC			Yes
Populus trichocarpa	1	A0A2K2BVT7_POPTR	Yes	No				Yes, except Domain A
Punica granatum	1	A0A218WH19_PUNGR	Yes	No				Yes
Quercus suber	1	none	No	No		A0A2P4HZ70_QUESU	Yes	No (E, F, G, H truncated)
Setaria italica	6	A0A368RDT7_SETIT	Yes	No	A0A368RDU3_SETIT A0A368RDZ6_SETIT A0A368RE07_SETIT	A0A368RDT4_SETIT A0A368RDT5_SETIT		Yes
Sorghum bicolor	2	A0A1B6PMR7_SORBI	Yes	No	A0A1Z5REK7_SORBI			Yes
Theobroma cacao	1	A0A061G5Z9_THECC	Yes	No				Yes
Trema orientale	1	A0A2P5D2T5_TREOI	Yes	No				Yes
Trifolium pratense	2	none	No	No		A0A2K3PP54_TRIPR A0A2K3P766_TRIPR	Yes	No (E, F, G, H truncated)
Vigna radiata var. radiata	1	A0A1S3VSZ1_VIGRR	Yes	No				Yes
Vitis vinifera	1	F6HUI3_VITVI	Yes	No				Yes
Zea mays	3	see Table S10	Yes	No	GRMZM2G007681_P01	A0A1D6KIX4_MAIZE A0A1D6KIX5_MAIZE A0A1D6KIY2_MAIZE		Yes
Total protein sequences:	96	Identified via the NRPD1 N-terminus motif						
**From distinct plant species:**	**51**							
**& without major truncations:**	**46**							

Table S12. Statistical tests performed on small RNA-seq data in Figure 3C

Pairwise comparisons using Wilcoxon rank sum test
data: log2RPKM.24 and Sample

	**Col-0**	**SucSul**	***nrpd1-3***	***nrpd1-47***	***nrpd1-49***	***nrpd1-50***	***nrpd1-51***	**Outcross**
**SucSul**	0.065	-	-	-	-	-	-	-
nrpd1-3	<2e-16	<2e-16	-	-	-	-	-	-
nrpd1-47	<2e-16	<2e-16	3.20E-07	-	-	-	-	-
nrpd1-49	<2e-16	<2e-16	0.053	0.035	-	-	-	-
nrpd1-50	<2e-16	<2e-16	<2e-16	<2e-16	<2e-16	-	-	-
nrpd1-51	<2e-16	<2e-16	3.30E-14	<2e-16	<2e-16	<2e-16	-	-
**Outcross**	0.432	0.018	<2e-16	<2e-16	<2e-16	<2e-16	<2e-16	-
nrpe1-11	<2e-16	<2e-16	<2e-16	<2e-16	<2e-16	<2e-16	<2e-16	<2e-16

P value adjustment method: holm

ns p > 0.01
* p <= 0.01
