# Supplementary material for: The NRPD1 N-terminus contains a Pol IV-specific motif that is critical for genome surveillance in Arabidopsis
Source: Nucleic Acids Res. 2019 Aug 2;47(17):9037–52. doi: 10.1093/nar/gkz618 (PMC6753494; doi:10.1093/nar/gkz618)
Supplement: gkz618_Supplemental_Files [file gkz618_supplemental_files.zip › Ferrafiat_2019_SupplData_gkz618.pdf]

SUPPLEMENTARY DATA

Supplementary figure legends

Figure S1. (A) The parental line for the genetic screen harbors a *SUC2::IR-SUL* transgene composed of the Arabidopsis *SUC2* (At1g22710) gene promoter driving transcription of a *Sulfur* gene (*SUL*, At4g18480) fragment cloned in an inverted repeat configuration. Forward and reverse segments of the *SUL* inverted repeat are separated by the *CHS* intron. This silencer construct produces *SUL* double-stranded RNAs (dsRNAs) that are diced into siRNAs, triggering degradation of endogenous *SUL* mRNAs. The inset photo shows the phenotype of a wild-type (WT) SucSul plant. Vascular bleaching in rosette leaves occurs because the SUL magnesium chelatase, which is knocked down, is required for chlorophyll biosynthesis. **(B)** WT *NRPD1* gene sequences and corresponding animo acid translations are shown, along with Sanger sequencing chromatograms, DNA base calls and predicted amino acid changes for each mutant: *nrpd1-47* (C56Y), *nrpd1-49* (G72E), *nrpd1-50* (C118Y) and *nrpd1-51* (W664*). For *nrpd1-48*, exons 2 and 3 of *NRPD1* are shown for both WT and mutant genotypes. The *nrpd1-48* mutation causes a splicing defect and frame shift leading to premature stop codons (solid black rectangles).

Figure S2. (A) Multiple alignment of NRPD1 proteins from diverse angiosperm species along with yeast RPB1 (Pol II, *S. cerevisiae*), Arabidopsis NRPB1 (Pol II, *A. thaliana*) and Arabidopsis NRPE1 (Pol V, *A. thaliana*), centered on the conserved "Domain A". **(B)** Small RNA blot analysis of *rdr2-2* rescue by RDR2-FLAG: small RNAs were size-separated, blotted and successively hybridized to probes for the Pol IV-RdDM targets *AtREP2* and *SIMPLEHAT2*, respectively, and then for miR160 (a loading control). **(C)** Transcripts from the *AtSN1* locus detected by semi-quantitative RT-PCR. **(D)** *Chop-PCR*: genomic DNA from WT SucSul D4, *nrpd1-48*, *nrpd1-49*, *nrpd1-50, nrpd1-51* and *nrpd1-3* plants was digested with the methylation-sensitive restriction enzyme *Hae* III, followed by PCR amplification of the *AtSN1* retroelement locus. Successful PCR indicated that the template DNA was methylated (protected), whereas weaker amplification indicated that little or no DNA methylation was present. "No digest" controls are PCR products from assays omitting the restriction enzyme. **(E)** Replicate co-immunopurification of the Pol IV complex with RDR2 (see Fig. 1D). Total protein input (left) and Anti-FLAG immunopurified protein (right) were separated on an SDS 4-20% gradient polyacrylamide gel and subjected to western blotting. Antibodies raised against the NRPD1 C-terminus or the NRPD2 N-terminus were used to successively detect these Pol IV catalytic subunits in protein fractions co-purified with RDR2-FLAG (middle panels), then a commercial anti-FLAG antibody was used to detect RDR2-FLAG (top panel). The faint band above NRPD2 (~133 kDa) in the third panel (*) corresponds to residual NRPD1 (~163 kDa) signal persisting after antibody stripping. Staining with Coomassie solution was used to evaluate protein loading in the input fractions. **(F)** Western blot to detect RDR2-FLAG protein immunoprecipitated from protein extracts of plants expressing either WT NRPD1 or the distinct *nrpd1* mutant variants (see Fig. 2C).

Figure S3. Analysis of 21 and 24 nt siRNAs at *pol IV* DMRs (see Fig. 3C). Boxplot quantification of 21 nt siRNAs (light blue) and 24 nt siRNAs (light purple) detected at hypo-DMRs identified in the comparison of

WT SucSul to the *nrpd1-51* (*pol IV*) mutant. The siRNA counts within each DMR were normalized by reads per kilobase per million mapped +1 (RPKM+1) and expressed on a Log_2 scale.

Figure S4. NRPD1 protein alignment and domain structure relative to mutations used in this study (see Fig. 4). ***Above:*** Domains A to H are conserved in multisubunit RNA polymerases (46–48, 57). Together, the bridge helix (BH) of "Domain F", trigger loop (TL) of "Domain G" and the extended C-terminal domain of tandem heptad repeats constitute a domain combination specific to the largest subunit of Pol II (shown here in *S. cerevisiae* RPB1 and *A. thaliana* NRPB1). The C-terminal domain containing WG/GW motifs and SQ-rich repeats is characteristic of the largest subunit of Pol V (shown here in *A. thaliana* NRPE1) (58). The "Defective in Chloroplasts and Leaves" (DeCL) domain forms part of the (otherwise distinct) C-terminal domains of the largest subunits of Pol IV and Pol V (NRPD1 and NRPE1, respectively). ***Middle:*** The Pol IV-specific motif uncovered by our study is evolutionarily conserved in NRPD1 from diverse plants, including *A. thaliana*, *Capsella rubella*, *Eutrema salsugineum*, *Theobroma cacao*, *Citrus* x *sinensis*, *Linum usitatissimum*, *Crocus sativus*, *Phaseolus vulgaris*, *Medicago truncatula*, *Eucalyptus grandis*, *Solanum lycopersicum*, *Setaria italica*, *Zea mays*, *Brachypodium distachyon*, *Amborella trichopoda*, *Ginkgo biloba* and *Pinus canariensis*. ***Below:*** Positions of the *A. thaliana* NRPD1 N-terminus missense mutations (*nrpd1-47*, *nrpd1-49* and *nrpd1-50*), of the premature stop codon mutation (*nrpd1-51*) and of the T-DNA insertion mutation (*nrpd1-3*) used in this study.

Figure S5. (A) Statistical analysis of *AtSN1* retroelement methylation determined by amplicon-based bisulfite sequencing (see Fig. 5B). Each boxplot shows CHH methylation percentage over the indicated *AtSN1* interval (i1, i2 or i3) and specified samples. Wilcoxon rank sum tests were performed to compare these interval methylation values amongst the WT SucSul, *nrpd1-50* and *nrpd1-51* samples. Asterisks are shown to annotate WT-mutant comparisons with $p < 0.05$; non-significant differences (ns) are those with $p > 0.05$. **(B)** Whole-genome bisulfite sequencing (WGBS) methylation profiles at *AtSN1* for the same samples as analyzed in Fig. 5B. **(C)** Metaplots of CHH methylation levels from WGBS (see Fig. 5C) for three TE superfamilies overlapping hypo-DMRs identified in the WT SucSul to *nrpd1-51* null mutant comparison: LTR/*Copia* elements, n = 82 (left); *MuDR* elements, n = 659 (middle); and *Helitron* elements, n = 1438 (right). The TE annotation boundaries are indicated as 5' and 3'-ends, with 2 kb of genomic context included upstream and downstream. ***Above:*** WT Control (black lines), *nrpd1-50* (orange lines) and *nrpd1-51* (red lines) mutants. ***Below:*** Metaplot comparison of the three control genotypes WT Col-0 (black lines), WT SucSul (blue lines) and WT outcross (purple lines).

Figure S6. Epigenomic profiles at TEs with residual methylation in *nrpd1-50*. CHH methylation is shown as green vertical lines in the first five tracks, with the y-axis representing percentage methylcytosine (1 = 100%, 0 = 0%; genomic strand is indicated by lines above/below the x-axis). The abundance of 24 nt siRNAs is displayed as purple vertical lines in the last five tracks, as reads per million mapped (RPM) on a Log_2 y-axis scale. WT SucSul, *nrpd1-50*, *nrpd1-51*, *nrpd1-3* and *nrpe1-11* (*pol V* null) data are shown for the following: **(A)** the *META1* family LTR/*Copia* element AT3TE76010, **(B)** the *HELITRONY3* family element AT2TE65130, **(C)** the *ATN9_1* family MuDR element AT3TE40900 and **(C)** the *soloLTR* region.

Figure S7. (A) DRM2-dependence of residual TE methylation in *nrpd1* mutants. *Chop-PCR*: Genomic DNA from WT Col-0, WT SucSul, *nrpd1-47*, *nrpd1-49*, *nrpd1-50*, *nrpd1-51, drm2* and each *nrpd1 drm2* double mutant combination was digested with the methylation-sensitive restriction enzyme *Hae* III followed by PCR at either the AT1TE14315 or AT1TE29060 locus. Successful PCR indicated that the template DNA was methylated (protected), whereas weaker amplification indicated that little or no DNA methylation was present. “No digest” controls are PCR products from assays omitting the restriction enzyme. **(B)** CMT3-independence of residual TE methylation in *nrpd1* mutants. *Chop-PCR*: WT Col-0, WT SucSul, *nrpd1-47*, *nrpd1-49*, *nrpd1-50*, *nrpd1-51, cmt3* and each *nrpd1 cmt3* double mutant combination was digested with *Hae* III followed by PCR, like in panel A above. **(C)** Pol V-dependence and RDR6-independence of residual TE methylation in *nrpd1-50. Chop-PCR*: Genomic DNA from WT Col-0, WT SucSul, *nrpd1-50*, *nrpd1-51* and the *nrpd1 nrpe1* (left panel) or *nrpd1 rdr6* (right panel) double mutant combinations was digested with *Hae* III followed by PCR, like in panel A above.

Supplementary tables

Table S01. Oligonucleotide primer and probe sequences

Table S02. MethylC-seq read data (summary)

Table S03. Bismark analysis from mapped MethylC-seq data

Table S04. Bisulfite conversion rates for MethylC-seq data

Table S05. Differentially methylated regions (DMRs) called using BSseq

Table S06. Total DMRs identified using WT SucSul as control

Table S07. Small RNA-seq read data (summary)

Table S08. Small RNA-seq read mapping

Table S09. Level of 24 nt reads measured in *pol IV* DMRs

Table S10. Largest subunits of Pol II, Pol IV and Pol V used for multiple alignments

Table S11. Detection of the Pol IV-specific motif in NRPD1 from other plant species

Table S12. Statistical tests performed on small RNA-seq data in Figure 3C

A. SucSul silencer system for the screen

B. Sequencing of *nrpd1* point mutations

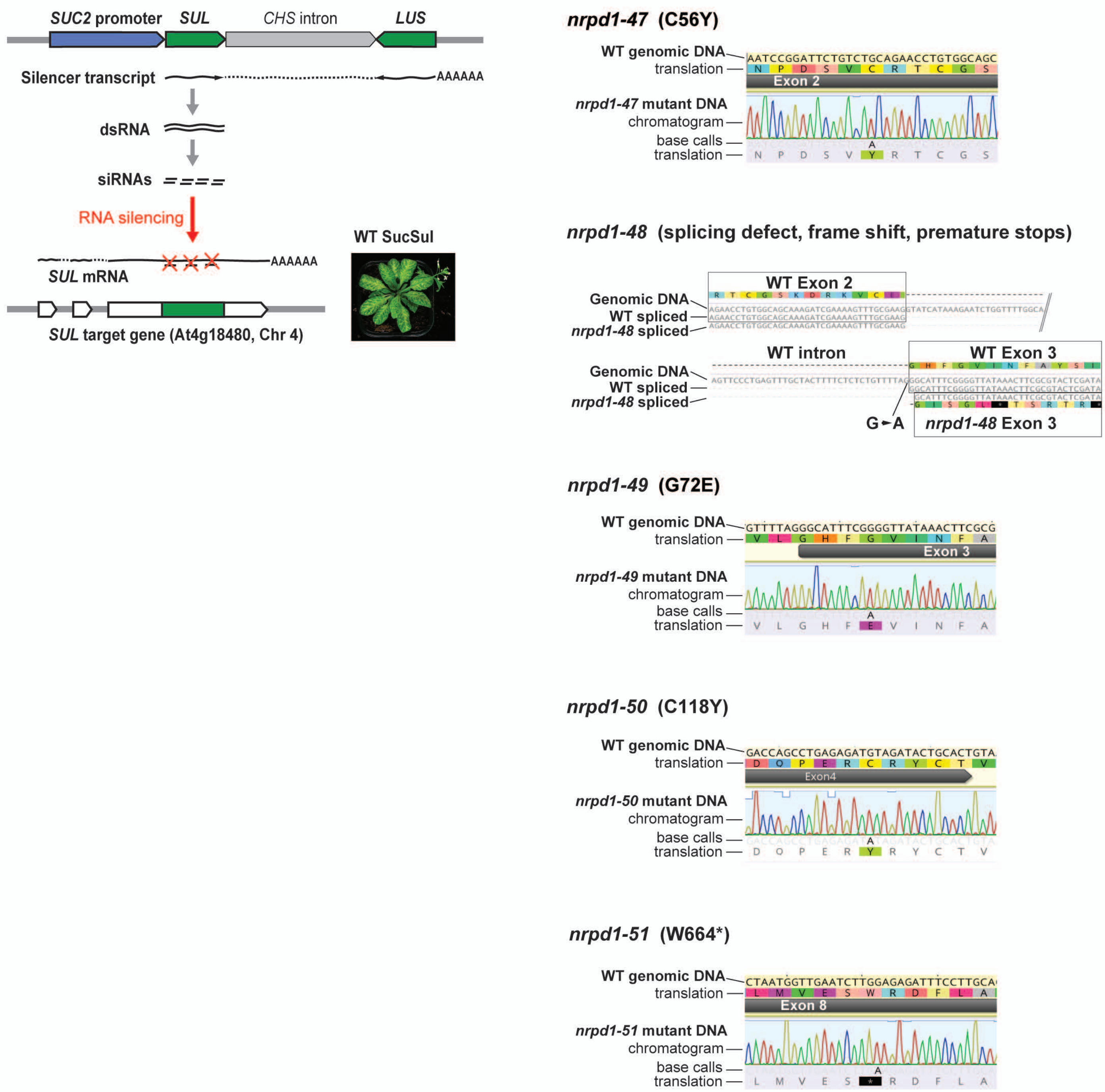

Ferrafiat et al., Supplementary Figure S1

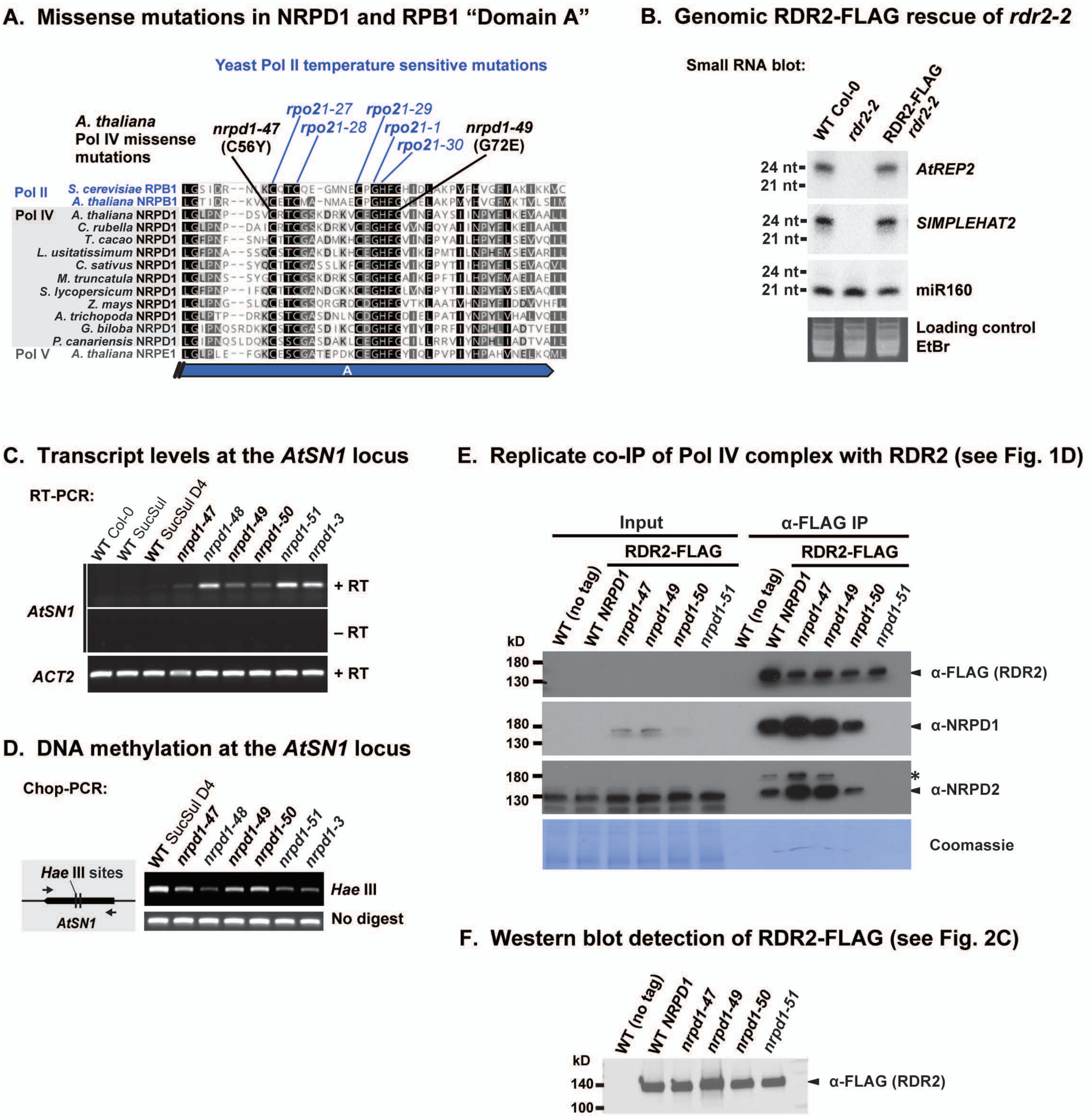

Ferrafiat et al., Supplementary Figure S2

Analysis of 21 and 24 nt siRNAs within *pol IV* DMRs (see Fig. 3C)

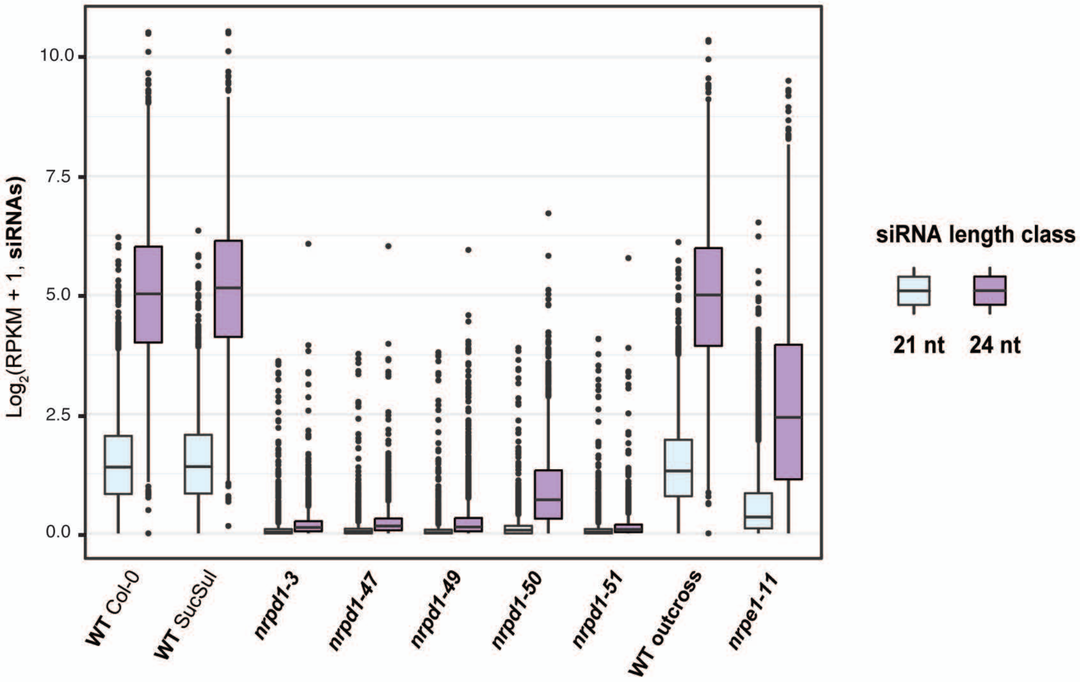

Ferrafiat et al., Supplementary Figure S3

NRPD1 protein alignment and domain structure relative to mutations used in this study (see Fig. 4)

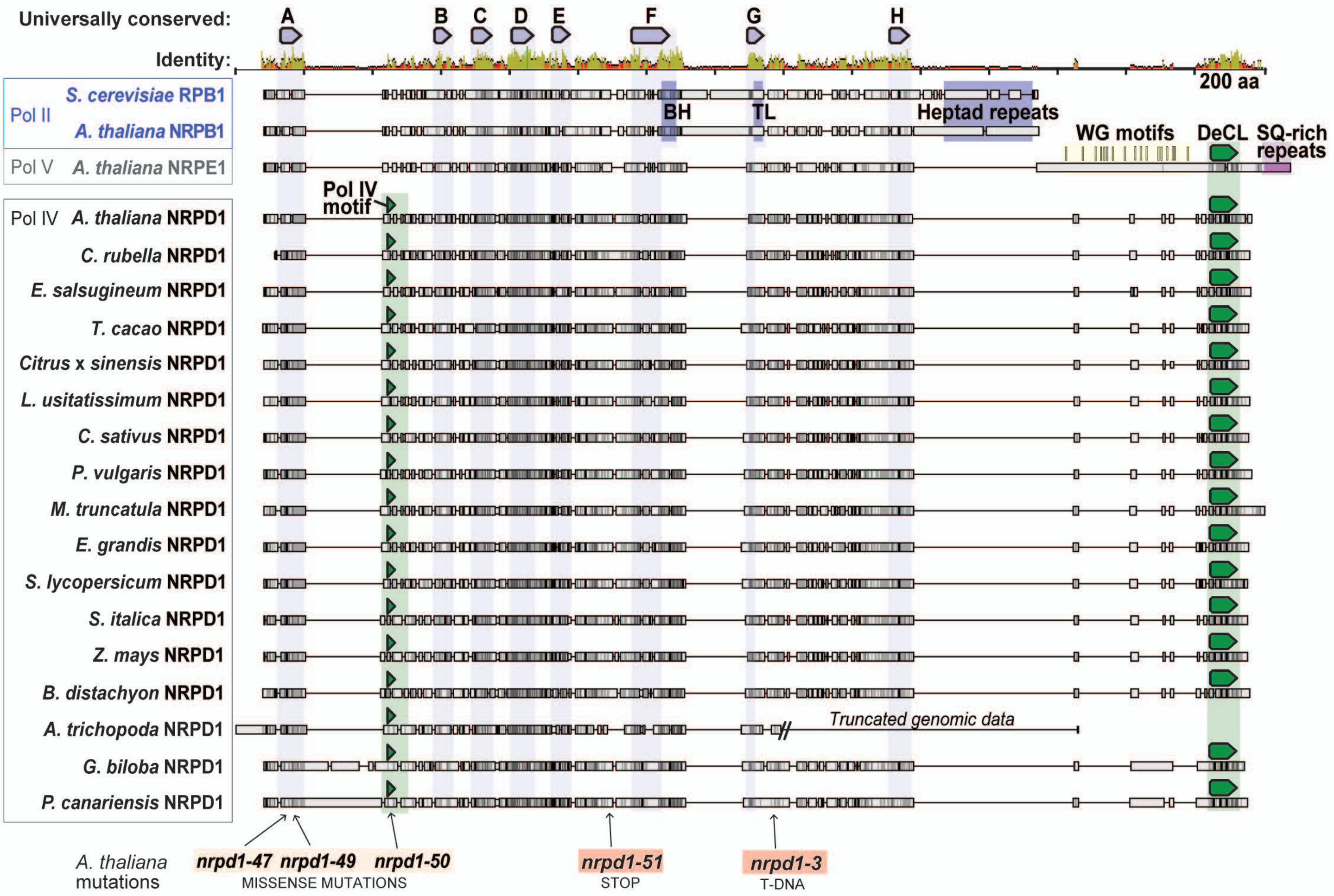

Ferrafiat et al., Supplementary Figure S4

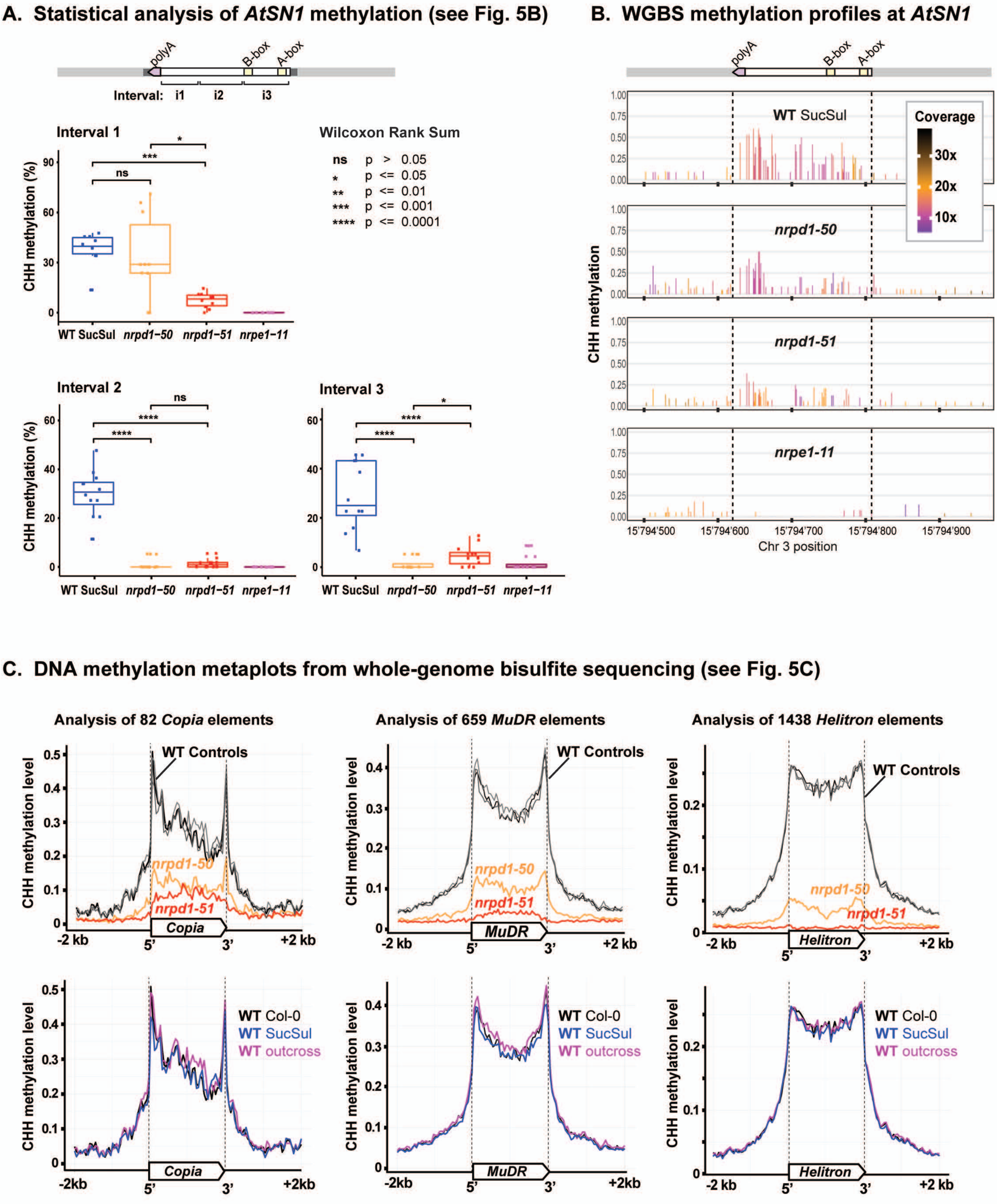

Ferrafiat et al., Supplementary Figure S5

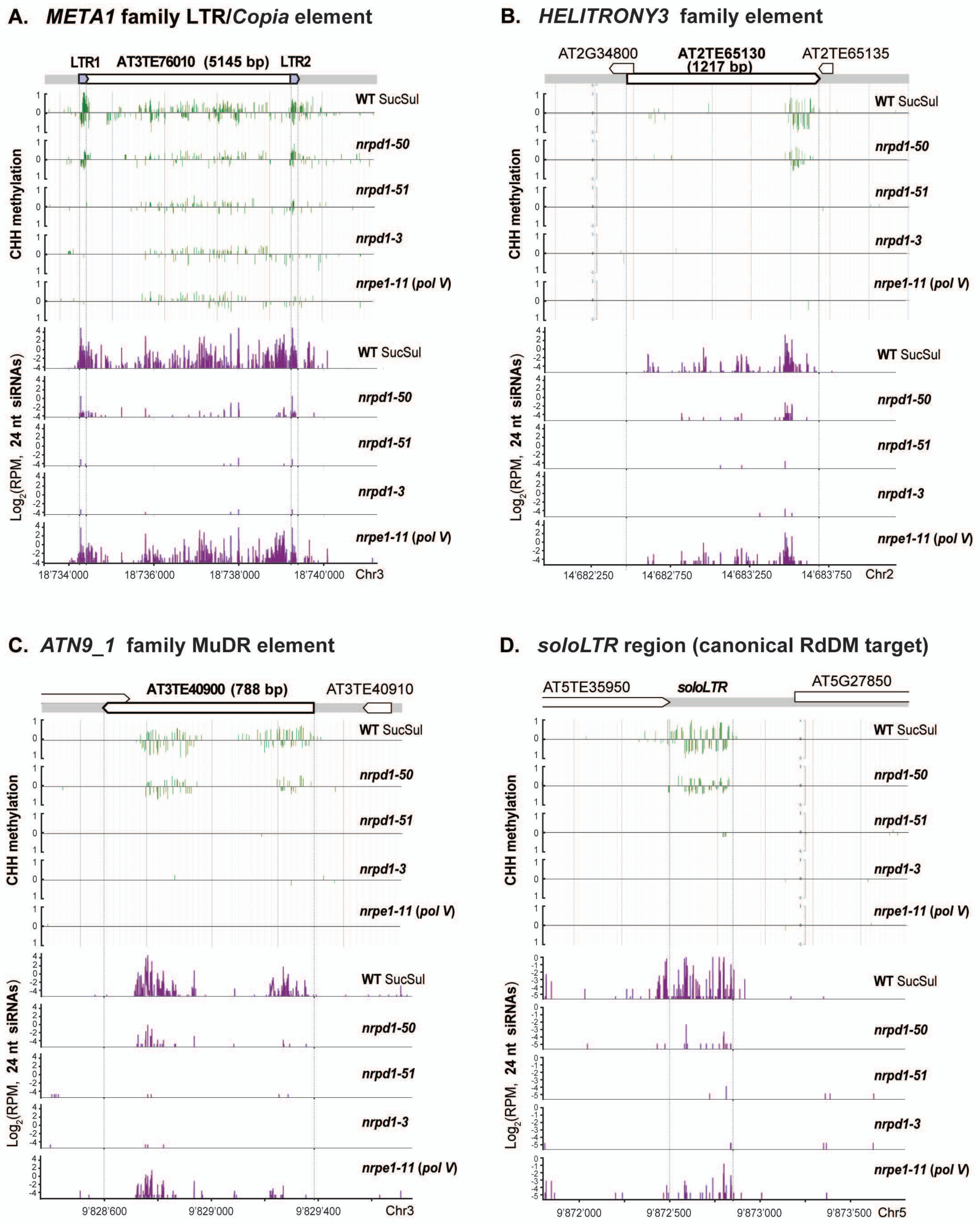

Ferrafiat et al., Supplementary Figure S6

A. DRM2-dependence of residual TE methylation in *nrpd1* mutants

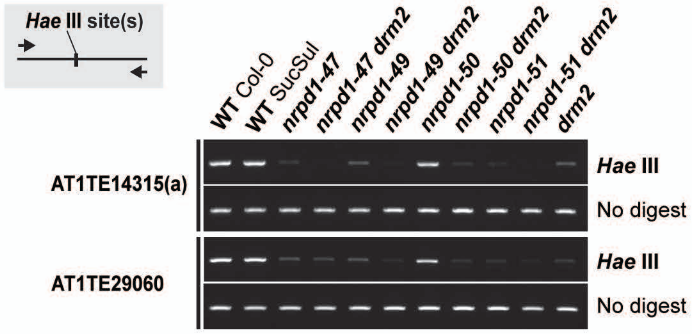

nrpd1-47, nrpd1-49, nrpd1-50 = hypomorphic mutants
nrpd1-51 = Pol IV null mutant
cmt3 = CMT3 null mutant
drm2 = DRM2 null mutant
nrpe1 = Pol V null mutant
rdr6 = RDR6 null mutant

B. CMT3-independence of residual TE methylation in *nrpd1* mutants

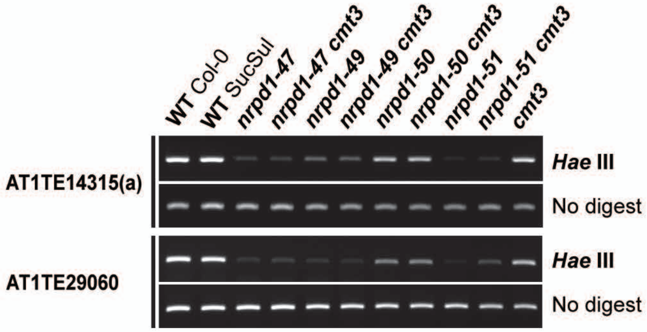

C. Pol V-dependence and RDR6-independence of residual TE methylation in *nrpd1-50*

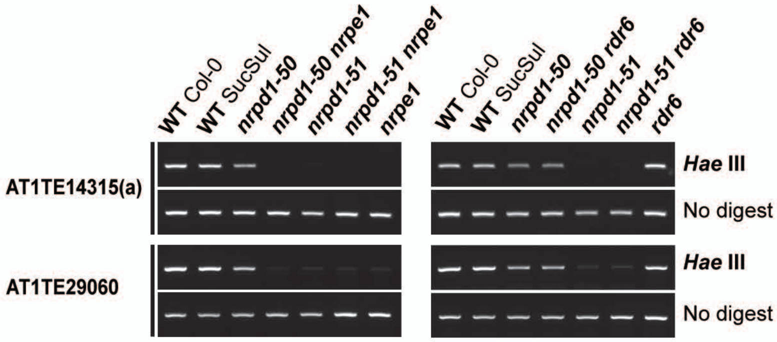

Ferrafiat et al., Supplementary Figure S7
